# Glioblastoma progression is hindered by melatonin-primed mesenchymal stromal cells through dynamic intracellular and extracellular reorganizations

**DOI:** 10.7150/thno.104143

**Published:** 2025-02-10

**Authors:** Laura Olmedo-Moreno, Concepción Panadero-Morón, Jesús María Sierra-Párraga, Rubén Bueno-Fernández, Emily S. Norton, Yolanda Aguilera, Nuria Mellado-Damas, Patricia García-Tárraga, Raquel Morales-Gallel, María Antequera-Martínez, Raúl V. Durán, Jaime Ferrer-Lozano, Germaine Escames, José Manuel García-Verdugo, Alejandro Martin-Montalvo, Hugo Guerrero-Cázares, Vivian Capilla-González

**Affiliations:** 1 Andalusian Molecular Biology and Regenerative Medicine Centre (CABIMER), CSIC-US-UPO-FPS, Seville, Spain.; 2 Neurosurgery Department, Mayo Clinic, Jacksonville, FL, USA.; 3 Cavanilles Institute of Biodiversity and Evolutionary Biology, University of Valencia, Valencia, Spain.; 4 Department of Pathology, Hospital Universitari i Politècnic La Fe, Valencia, Spain.; 5 Biomedical Research Center, Health Sciences Technology Park, University of Granada, 18016 Granada, Spain.; 6 Centro de Investigación Biomédica en Red sobre Enfermedades Neurodegenerativas (CIBERNED), Madrid, Spain.; 7 Centro de Investigación Biomédica en Red de Diabetes y Enfermedades Metabólicas Asociadas (CIBERDEM), Madrid, Spain.

**Keywords:** brain tumor, glioma, GBM, MSCs, mesenchymal stem cell, cell therapy, melatonin, cancer growth, cancer invasion, mouse xenograft

## Abstract

**Rationale:** Glioblastoma (GBM) is the most fatal form of brain cancer and its treatment represents a persistent challenge. Mesenchymal stromal cells (MSCs) have been explored as therapeutic tools in cancer management owing to their tumor-homing abilities. However, their clinical application is limited due to the controversial role of MSCs in carcinogenesis. This study investigates how MSCs influence tumor behavior and explores the synergistic anticancer effects in combination with melatonin (Mel).

**Methods:** Orthotopic and subcutaneous GBM xenograft mouse models were used to assess the antitumor effect of Mel pre-treated MSCs (MSC^Mel^). Histological, immunohistochemical, and ultrastructural analyses were conducted to identify phenotypic changes in tumors. Through a set of *in vitro* assays, including direct and indirect co-cultures, dynamic single-cell tracking and tumorsphere assay, we explored the impact of MSC^Mel^ on primary and non-primary GBM cells. Transcriptomic profiling was used to identify genes and pathways modulated by this synergistic therapy.

**Results:** MSC^Mel^ delayed tumor growth in mice and increased collagen deposition. Additionally, MSC^Mel^ showed enhanced capacity to prevent GBM cell migration compared to untreated MSCs. Molecular analysis identified genes and proteins related to cell migration, cytoskeletal dynamics and extracellular matrix remodeling in GBM cells exposed to MSC^Mel^, including reduced vimentin expression. Finally, a genetic signature associated with the clinical outcomes of GBM patients was identified.

**Conclusions:** Our study demonstrates that melatonin enhances the anticancer properties of MSCs, providing new insights into their interaction with GBM cells and tumor environment. These findings offer valuable guidance for advancing MSC-based therapies in clinical practice.

## Introduction

Glioblastoma (GBM) is the most common and aggressive primary brain tumor in adults, affecting people of any age, including children. The first-line treatment for patients with GBM is surgical resection, followed by radiotherapy and chemotherapy 1. However, the recurrence of GBM is almost inevitable due to its highly infiltrative nature, leading to a poor prognosis with a median survival of 12-15 months 2. This scenario highlights the urgent need to develop more effective therapies for GBM patients.

Mesenchymal stem/stromal cells (MSCs) are adult stem cells with self-renewal and multilineage differentiation potential. These cells exhibit potent paracrine properties that can modulate multiple cellular processes, including cell survival, inflammation, apoptosis, and angiogenesis, among other actions 3. Importantly, MSCs possess inherent tropism towards injury sites, making these cells a valuable tool for cell-based therapies and regenerative medicine. Similar to damaged tissues, the tumor environment produces chemoattractant signals that promote the recruitment of MSCs, enabling them to closely interact with cancer cells 3. Although previous reports suggesting that MSCs exert tumor-promoting effects, several studies have revealed that MSCs can act as potent tumor suppressors 4-14. For instance, a seminal report demonstrated that MSCs inhibit human glioma growth through downregulation of the PDGF/PDGFR axis, which plays a key role in tumor angiogenesis 4. Similar antiangiogenic properties were described after MSC inoculation in a mouse melanoma model 5. Other studies have genetically engineered MSCs to overexpress TNF-related apoptosis-inducing ligand (TRAIL) or bone morphogenetic protein 4 (BMP4), showing strong anti-tumor activity in brain cancer 6,7. Furthermore, incredible progress in engineering MSCs into Trojan horses capable of delivering anti-cancer drugs to specific target sites, exploiting their inherent tumor tropism, has been achieved during the last decade. Relevant studies have demonstrated that MSCs carrying the oncolytic adenovirus ICOVIR-5 (Celyvir) can serve as therapeutic vehicles, exerting beneficial effects in mice and dogs with tumors in the nervous system 8,9. Importantly, the Celyvir strategy has been translated into clinical studies involving patients with metastatic and refractory solid tumors, showing positive safety results 10. However, the success of MSC-based therapies depends on numerous factors that can potentially reduce the efficacy of transplanted cells, thus resulting in modest outcome improvements. Among these factors, the specific condition of the tumor environment is a major limiting aspect. Many tumors grow in a niche characterized by inflammation, hypoxia, oxidative stress, and low nutrient availability 11, which can reduce the survival of grafted MSCs. Therefore, the use of strategies combining MSCs and cytoprotective agents should be considered in cell therapy to achieve maximum clinical benefits.

One of the most common approaches to potentiate the therapeutic properties of MSCs is the preconditioning with different factors, including cytokines and cytoprotective agents 12. In this context, melatonin (Mel) has been used to efficiently promote the therapeutic functions of MSCs in different disease models. Melatonin is a natural hormone that acts as a potent free radical scavenger with antioxidant and anti-inflammatory activities, thus promoting cytoprotective effects. MSCs pre-treated with Mel (10 μM) prior to implantation exhibited better effects on recovering liver function than untreated MSCs, by enhancing hepatic engraftment after tail vein injection in a mouse liver fibrosis model 13. Similarly, the preconditioning with 5 μM Mel increased the number of grafted MSCs into the brain in a rat model of Alzheimer's disease, improving cognitive function as compared to untreated MSCs 14. Paradoxically, melatonin also exhibits cytotoxic and anti-proliferative effects in cancer cells, as demonstrated in animal models and cell cultures 15-20. For instance, mice bearing head and neck squamous carcinoma (HNSCC) xenografts exhibited a reduction in tumor growth when treated intratumorally with high concentrations of Mel 15,16. Therefore, the dual actions of melatonin as a cytoprotective and anti-tumor agent make this molecule an interesting component in cell-based therapies against cancer.

This study provides new insight into the mechanisms of interactions between MSCs and GBM cells using a combination of *in vitro* and *in vivo* models. In addition, we investigate how the pre-treatment of MSCs with Mel modulates their anticancer properties. Transcriptomic profiling and protein expression analysis uncover effects of MSCs on matrix remodeling, immune landscape and cytoskeletal rearrangements, which were potentiated by Mel pre-treatment. Our findings not only shed light on the complex dynamics between MSCs and GBM cells, but also unveil Mel pre-treatment as an effective strategy to enhance MSC anti-cancer actions, offering a novel avenue in cancer therapy.

## Materials and methods

### Cell culture

Human MSCs (#PCS-500-011, ATCC; Middlesex, United Kingdom) and GBM cells (U87MG, #HTB-14; T98G, #CRL-1690; LN-18, #CRL-2610, LN-229, #CRL-2611; ATCC) were cultured in growth media composed of Dulbecco's Modified Eagle Medium (DMEM; Life Technologies, Carlsbad, CA), supplemented with 10% fetal bovine serum (FBS), 1% penicillin-streptomycin and Glutamax or L-glutamine. The primary GBM cell line GBM1A was originally derived by Dr. Angelo Vescovi and colleagues 21. GBM1A cells were cultured on laminin coated flasks (0.01 mg/mL) (R&D systems, Minneapolis, MN, USA) in growth media composed of DMEM/F12 (ThermoFisher Scientific, Madrid, Spain), supplemented with Gem21 Neuroplex (Gemini Bio-products, Weste Sacramento, CA, USA), 1% penicillin-streptomycin, 20 ng/mL of human epidermal growth factor (*hEGF*; PeproTech, Rosemont, IL, USA) and 20 ng/mL of human Fibroblastic Growth Factor (hFGF; PeproTech, Rosemont, IL, USA). Cells were incubated at 37 °C in a 20% O_2_ and 5% CO_2_ humidified atmosphere. Media were changed every 2-3 days. The CM-DiI fluorescent dye (C7000; ThermoFisher Scientific) and the CMFDA fluorescent dye (C7025; ThermoFisher Scientific) were used to label different cell types in direct co-culture assays. For Mel pre-treatment, MSCs were cultured for 24 h in growth media containing 25 µM Mel (Fagron; Barcelona, Spain), which was determined as the optimal concentration (i.e., minimal concentration promoting cytoprotective effects) (**Figure S1**). The exposure time was chosen based on previous reports 14,22,23. MSC phenotyping after Mel pre-treatment was performed to evaluate possible changes in cell identity (**Figure S1**).

### Flow cytometry

Cultured cells were harvested and incubated for 20 min with the appropriated primary antibodies in the dark at room temperature (see **Table S1** for antibody information). After primary antibody incubation, cells were washed with PBS and centrifuged at 2000 rpm for 5 min. For Ki67 staining, we used a PE Mouse Anti-Ki67 Set (556027, BD Biosciences), according to manufacturer's protocol. Briefly, cells were fixed and permeabilized with ethanol at -20 ºC for 2 h and incubated with primary antibody. For tumorspheres Ki67 staining, GBM cells were pre-stained with CMFDA fluorescent dye (C7025; ThermoFisher Scientific). CMFDA+ and Ki67+ GBM cells were quantified. All samples were analyzed using an LSRFortessa X-20 flow cytometer (BD Biosciences, San Diego, CA, USA) and the BD FACSDiva software (BD Biosciences).

### Senescence assay kit

Senescence-associated β-galactosidase activity was detected on attached MSCs (treated or not with melatonin) using a Senescence Assay Kit (ab228562, Abcam, Cambridge, UK), according to manufacturer's protocol. After proper staining, cells were collected by trypsinization, washed and analyzed for FITC signal using an LSRFortessa X-20 flow cytometer (BD Biosciences) and the BD FACSDiva software (BD Biosciences).

### Colony-forming unit assay

Cells were plated onto 100 mm dishes at a low density (100 cells per dish) and cultured for 14 days in growth media. Then, cells were fixed with 4% paraformaldehyde (PFA) and stained with 0.5% cresyl violet to analyze the number and size of the colonies using ImageJ software (version 1.4r; National Institute of Health, Bethesda, MD, USA).

### Cell viability assay

Cell viability was evaluated using the AlamarBlue™ Cell Viability Reagent (Life Technologies) or the Cell Proliferation Kit I (MTT; Roche, Spain), according to the recommendations of the manufacturer. For AlamarBlue™ assay, optical density was determined after 4 h of incubation at 570 nm with a reference wavelength of 600 nm, using a Varioskan Flash spectrophotometer (ThermoFisher Scientific). For MTT assay, optical density was determined after 4 h of incubation at 575 nm with a reference wavelength of 690 nm.

### Western blot

Samples were lysed in RIPA buffer (Sigma-Aldrich, Madrid, Spain), containing 1X protease inhibitor (Roche Diagnostics, Mannheim, Germany) and 1X phosphatase inhibitor cocktail (ThermoFisher Scientific). Electrophoresis was run using acrylamide gels (10-15% acrylamide concentration, depending on protein size) followed by protein transfer to nitrocellulose membranes. Western blots were performed according to standard methods, which involved a blocking step and then incubation with a primary antibody of interest overnight at 4 ºC (**Table S1**), followed by incubation with a horseradish peroxidase-conjugated secondary antibody, and enhanced chemiluminescence. Blots were quantified with ImageJ software and the bands of interest were normalized to GAPDH or β-actin staining.

### Oxygen consumption and extracellular acidification rates

Mitochondrial bioenergetics on MSCs were measured using an XF24 Extracellular Flux Analyzer (Agilent Technologies, Santa Clara, CA, United States). After 24 h of treatment with melatonin, cells were washed with Seahorse assay media supplemented with 10 mM glucose, 1 mM pyruvate, and 2 mM glutamine, pH 7.2 (Agilent Technologies). Cells were incubated in a CO_2_-free incubator at 37 °C for 1 h. Then, OCR and ECAR were determined in basal conditions and through consecutive injections of oligomycin (1 μM) at min 24, carbonyl cyanide 4-(trifluoromethoxy) phenylhydrazone (FCCP; 1 μM) at min 48, rotenone (Rot; 5 μM) at min 72, and antimycin A (AA; 1 μM) at min 96.

### Lentivirus production and transduction

pLenti CMV GFP Puro (658-5) (Addgene plasmid, #17448; kindly provided by Dr. Eric Campeau & Dr. Paul Kaufman) 24 and pLV[Expression]-mCherry:T2A:Hygro-EF1A>Luc2 (Addgene plasmid, #174665; kindly provided by Dr. Eli Bar) lentivirus plasmids were produced by transfecting human embryonic kidney cells (HEK293T; #CRL-3216, ATCC). 10·10^6^ HEK293T cells were seeded in a 100 mm culture dish using 6 mL of DMEM media with 5% FBS and no antibiotics. Cells were incubated at 37 °C in a 20% O_2_ and 5% CO_2_ humidified atmosphere during 24 h. HEK293T transfection was performed using the transfection reagents lipofectamine 3000 and P3000 reagent (ThermoFisher Scientific). OptiMEM reduced-serum medium (ThermoFisher Scientific) was used to minimize serum interference. Packaging plasmids psPAX2 (Addgene plasmid, #12260) and envelope plasmid pMD2.G (Addgene plasmid, #12259) were kindly provided by Dr. Didier Trono. After 6 h of incubation with GFP and m-Cherry plasmids, media was replaced with 6 mL of DMEM with 10% FBS and no antibiotics. The viral supernatant was collected after 24- and 48 h post-transfection and subjected to centrifugation at 300 g for 5 min at 4 ºC. Supernatant was filtered through a 0.45 μm pore size filter. For lentivirus concentration, Lenti-X-Concentrator (Takara Bio, Shiga, Japan) was used at a ratio 3 media:1 concentrator. After an incubation of 30 min at 4 ºC, the samples were centrifuged 1,500 g at 4 ºC and the white pellet was collected and diluted in 1X PBS. For viral transduction of GBM cells with the m-Cherry lentivirus, 8·10^5^ cells were seeded in a T-25 flask. After adherence, U87 cells were transduced for 48 h with 4 μg/mL of polybrene and 2 μL of m-Cherry lentivirus. GBM1A cells were transduced for the same duration with 2 μg/mL of polybrene and 5 μL of m-Cherry lentivirus. For viral transduction of MSC with the GFP lentivirus, 100,000 cells were seeded in a 6 well plate. After their adherence, 1 μL of GFP virus and 10 ng/mL FGF* (*Peprotech*)* were added. To facilitate transduction, MSCs were centrifuged at 1,200 g for 1 h at room temperature and incubated for 72 h. The media was then removed and replaced with fresh media. One day after, the m-Cherry and GFP positive cells were selected through the use of 150 μg/mL hygromycin B (InvivoGen, San Diego, CA, USA) or 1 μg/mL puromycin (InvivoGen), respectively. Antibiotics were changed every other day for a period of 10 days.

### Subcutaneous xenograft model

Eleven-week-old athymic nude mice (Charles River Laboratories; Barcelona, Spain) were anesthetized with 2% isoflurane. Then, animals were injected subcutaneously in the flanks with 225 μL of DMEM:matrigel (1:2) mixture, containing 1·10^6^ U87 cells, with or without 1·10^6^ MSC or MSC^Mel^. Mice were monitored daily and tumors were measured with a digital caliper rule every 5 days. Tumor volume was estimated using the formula: (minor diameter^2^ × major diameter)/2 25,26. All animals were sacrificed 30 days after tumor inoculation. Mice were subjected to intracardiac perfusion with 4% PFA, using a peristaltic pump. Tumor masses were resected and weighted. Tumor volume at the endpoint was determined by the Water Displacement Method and density was estimated from weight and volume (g/mL). Fixed tumors were embedded in paraffin, sectioned in 8 μm-thick slices and processed for histological and immunohistochemistry analysis. Subcutaneous xenograft procedures were approved by the CABIMER Ethics Committee for Animal Experimentation, and complied with national and European Union legislation (Spanish RD 53/2013 and EU Directive 2010/63) for the protection of animals used for scientific purposes.

### Orthotopic xenograft model

Six-week-old athymic nude mice (Jackson Laboratories, Bar Harbor, ME, USA) were used for the study. Intracranial injections were performed as described previously 27,28. Mice were anesthetized with 2.5% isoflurane and immobilized in a stereotaxic apparatus. A mixture of 3.5·10^5^ m-Cherry-Luc U87 cells with or without 3.5·10^5^ GFP MSC or MSC^Mel^ were resuspended in 2 μL of PBS and injected intracranially into the right hemisphere (coordinates from bregma in mm: Y: 1.00; X:1.75 and Z:3.2). Injection was performed automatically at 0.5 μL/min rate. After an extra min, the injection needle was slowly withdrawn and skin incision was closed with surgical glue. For a correct animal recovery, buprenorphine (10 mg/kg) was used as pre- and post-operative analgesic. Tumor progression monitoring was performed by bioluminescence imaging twice per week over four weeks. Briefly, mice were injected intraperitoneally with D-Luciferin potassium salt (XenoLight, Perkin Elmer, Waltham, MA, USA) at a dose of 150 mg/kg. After 10 min, mice were anesthetized with 2.5% isoflurane and placed in prone position in the IVIS Spectrum System (Perkin Elmer) for *in vivo* imaging. Regions of interest (ROIs) were selected around the tumor for analysis (photons/sec/cm^2^/sr). Mice were euthanized 4 weeks after surgery and perfused with 0.9% saline followed by 4% PFA using a peristaltic pump. Fixed brains were dehydrated and embedded in paraffin before sectioning on a rotary microtome at 5 μm thickness. Slides were deparaffinized and rehydrated prior to histological staining and immunostaining. Then, slides were imaged using a histology slide scanner (Leica Aperio AT2). Orthotopic xenograft procedures were approved by Mayo Clinic Institutional Animal Care and Use Committee in accordance with National Institutes of Health guidelines.

### Transwell assay

Migration of GBM cells (U87) in response to the presence of MSCs or MSC^Mel^ was assessed using indirect co-cultures (without cell-cell contact). MSCs or MSC^Mel^ were seeded in a 24-well plate and allowed to attach. Separately, GBM cells were seeded in a hanging cell culture inserts (8 µm pore size; CellQuart; Northeim, Germany) and allowed to attach. The day after, inserts with GBM cells were transferred to the wells containing MSCs or MSC^Mel^. Co-cultures were maintained for 24 h in FBS-free media supplemented with 1% penicillin-streptomycin. Then, inserts were rinsed in PBS, fixed in 4% PFA and stained in 0.5% cresyl violet to evaluate migration of GBM cells through the membrane pores. To quantify the number of migrated cells, 10 random fields per membrane were imaged using a Leica DM6000B microscope equipped with a DFC390 camera (Leica, Wetzlar, Germany).

### Livecyte microscopy

A 12-well glass-bottom plate was coated with Poly-L (R&D systems) and incubated for 1 h at 37 ºC. After the incubation, Poly-L was aspirated and the plate was coated with 0.01 mg/mL laminin for 1 h at 37 ºC. After laminin aspiration, 6.3·10^5^ GBM cells were seeded with or without 1.8·10^4^ MSC or MSC^Mel^ and incubated overnight. The plates were placed onto the Phasefocus Livecyte 2 (Phasefocus Limited, Sheffield, UK) 20 min prior to image acquisition to allow the incubator to equilibrate. Phase and fluorescence small image area of two fields per well were taken every 10 min over 48 h at 10x magnification to study the behavior of live cells in a 2D system.

### GBM tumorspheres

To evaluate 3D migration, GBM tumorspheres were generated by placing 2.1·10^4^ cells in a 20 μL hanging drop with growth media. For 3D co-cultures, GBM cells combined with MSCs or MSC^Mel^ (3:1 ratio) were used. After 72 h, tumorspheres were transferred to pre-coated wells. Fibronectin (Sigma-Aldrich) or laminin (R&D systems) pre-coating was used to evaluate migration, according to cell preferences. Matrigel (Corning; Amsterdam, Netherlands) pre-coating was used to evaluate invasion. Images of the tumorspheres were captured every 24 h and analyzed using the ImageJ software. Migration and invasion area were defined as the entire area in which the cells moved on the scaffold. Maximum distance represents the measurement of the furthest distance traveled by cells on the scaffold. Values at time 0 were subtracted from each measurement.

### RNA isolation and sequencing

GBM cells (U87) were cultured either alone or with MSC or MSC^Mel^ using the transwell system (MSCs were seeded in the upper chamber) for 24 h. Then, RNA was isolated from GBM cells using the RNeasy Mini kit (QIAGEN, Hilden, Germany), according to the manufacturer's instructions. RNA concentration was quantified using a Qubit™ RNA HS Assay kit and RNA quality was analyzed using a Bioanalyzer 2100 (Agilent Technologies). Briefly, 150 ng of total RNA from each sample was used to obtain the libraries following the TruSeq Stranded mRNA protocol of Illumina (Illumina Inc). The quality control of the generated libraries was carried out with the Bioanalyzer DNA High sensitivity chip. Paired-end sequencing was performed using NextSeq 500 Mid Output and 2x75 pb (Illumina Inc), generating more than 24 Gbases. Sequencing quality, assessed with BaseSpace Onsite software (v. 3.22.91.158, Illumina), was high (89% ≥Q30), with the FastqGeneration app yielding 24 million of paired reads (passing filter) per sample (fastq format). Differential analysis for all comparisons and raw counts were generated with DRAGEN and DESeq2 from Illumina, respectively. Hallmark analysis of the gene counts showing enriched gene sets related to different biological processes was performed with *GSEA.* DEG with a p < 0.05 and Log2 of fold-change ≥ 1 or ≤ -1 in each comparative set were included in the analysis. Canonical pathway analysis of the DEG was performed using Ingenuity Pathway Analysis (IPA) software from Qiagen. Venny diagram was generated using the open-source online tool Venny 2.1.0. The PANTHER resource was used to identify unique genes of each comparison. SRplot web server was used to generate the chord-plots and the circular cluster heatmap. The gene signature obtained of differentially expressed genes in the comparation GBM+MSC vs. GBM+MSC^Mel^, employed to categorize distinct clusters of The Cancer Genome Atlas (TCGA) GBM patients using RNA-seq data, was analyzed through the use of R studio (2023). RNA sequencing was performed in the Genomic Unit of CABIMER. Raw data are accessible in Gene Expression Omnibus (GEO) database, with the accession number GSE268582.

### Cytokines bio-plex assay

The secretome of GBM cells alone or co-cultured with MSCs or MSC^Mel^ during 24 h was assayed using the Bio-Plex Pro™ Human Cytokine assay kit (Bio-Rad Laboratories, Hercules, CA, USA), according to the manufacturer's instructions. Analyzed cytokines included Eotaxin, G-CSF, GM-CSF, IFN-γ, IL-1β, IL-1ra, IL-2, IL-4, IL-5, IL-6, IL-7, IL-8, IL-9, IL-10, IL-12 (p70), IL-13, IL-15, IL-17A, IP-10, MCP-1 (MCAF), MIP-1α, MIP-1β, PDGF-BB, RANTES, TNF-α, and VEGF. Cytokines were analyzed using the Bio-Plex 200 system (Bio-Rad Laboratories) and raw data was processed using the Bio-Plex Manager software version 4.1.1 (Bio-Rad Laboratories).

### Glioblastoma specimen collection and processing

Glioblastoma samples were obtained through surgical resection of newly diagnosed untreated patients (15 patients) at the Hospital Universitari i Politècnic la Fe after informed consent. Survival data were available for 11 patients. Samples were fixed in 4% PFA and subsequently embedded in paraffin for sectioning. Glioblastoma sections were organized in tissue microarrays (TMA), with 3-4 tumor spots from each sample, representing morphologically diverse zones from the tumor. Sections were deparaffinized in xylene and rehydrated in decreasing concentrations of ethanol prior to histological and immunostaining analysis.

### Histology and immunostaining

Hematoxylin and Eosin and Sirius Red stainings were carried out following standard protocols. Orientation analysis of Sirius Red stained samples was performed with the ImageJ plugin OrientationJ. For immunohistochemical staining, sections were incubated in the appropriated antigen retrieval, followed by blocking solution for 1 h at room temperature, and then incubated overnight at 4 ºC with primary antibodies (see **Table S1** for antibody information). Then, sections were washed and incubated with the appropriate secondary biotinylated antibodies, followed by incubation with the avidin-biotin complex (Vectastain ABC-HRP Kit, Peroxidase - IgG; Vector Laboratories, Burlingame, CA, USA) and then treated with diaminobenzidine-nickel substrate (DAB-nickel; Vector Laboratories). For immunofluorescence, Alexa Fluor conjugated secondary antibodies were used and nuclei were counterstained with DAPI or Hoechst. TUNEL assay was performed to detect cell death with the In-Situ Cell Death Detection Kit, TMR red (#12156792910, Roche, Germany). MitoTracker Orange CMTMRos dye (80 nM; M7510 ThermoFisher Scientific) was used to stain mitochondria. All samples were examined under a Leica DM6000B microscope equipped with a DFC390 camera (Leica) and Leica Thunder Imager microscope. Fluorescence signal was quantified using ImageJ software. Visual score analysis of Iba1 was performed by two independent blinded observers and signal intensity in the whole section was classified as negative (score 0), weak (score 1), moderate (score 2), strong (score 3), very strong (score 4), and extremely strong (score 5).

### Transmission electron microscopy

Fixed tumors were postfixed in 2.5% glutaraldehyde, rinsed in PBS and cut into 200 µm sections using a VT 1000M vibratome (Leica). Then, sections were processed for electron microscope, as previously described 29. Briefly, sections were postfixed in 2% osmium tetroxide, dehydrated in ethanol, stained in 2% uranyl acetate and embedded in araldite (Durcupan, Fluka BioChemika, Ronkonkoma, NY, USA). Ultrathin sections (60-70 nm) were cut with a diamond knife, stained with lead citrate, and examined under a Spirit transmission electron microscope (FEI Tecnai, Hillsboro, OR, USA).

### Statistical analysis

Data were expressed as the means ± SEM and analyzed using GraphPad Prism 10 software (GraphPad Software Inc., San Diego, CA, USA). Parametric ANOVA followed by a Tukey´s, Dunnett's or Šidák *post-hoc* test was performed to compare more than two experimental groups. Comparisons between two experimental groups were performed with Student's t-test. Repeated-measures ANOVA was applied when appropriated. Log-rank test was performed to determine differences between the experimental groups in the probability of an event at any time point (i.e., tumor size or survival). Log-rank (Mantel-Cox) test was used to analyze survival curves of patient clusters according to TCGA database. All differences were considered significant at a *p*-value < 0.05.

## Results

### Melatonin enhances the viability of human MSCs under tumor-like adverse conditions without altering their identity

**Figure 1:**
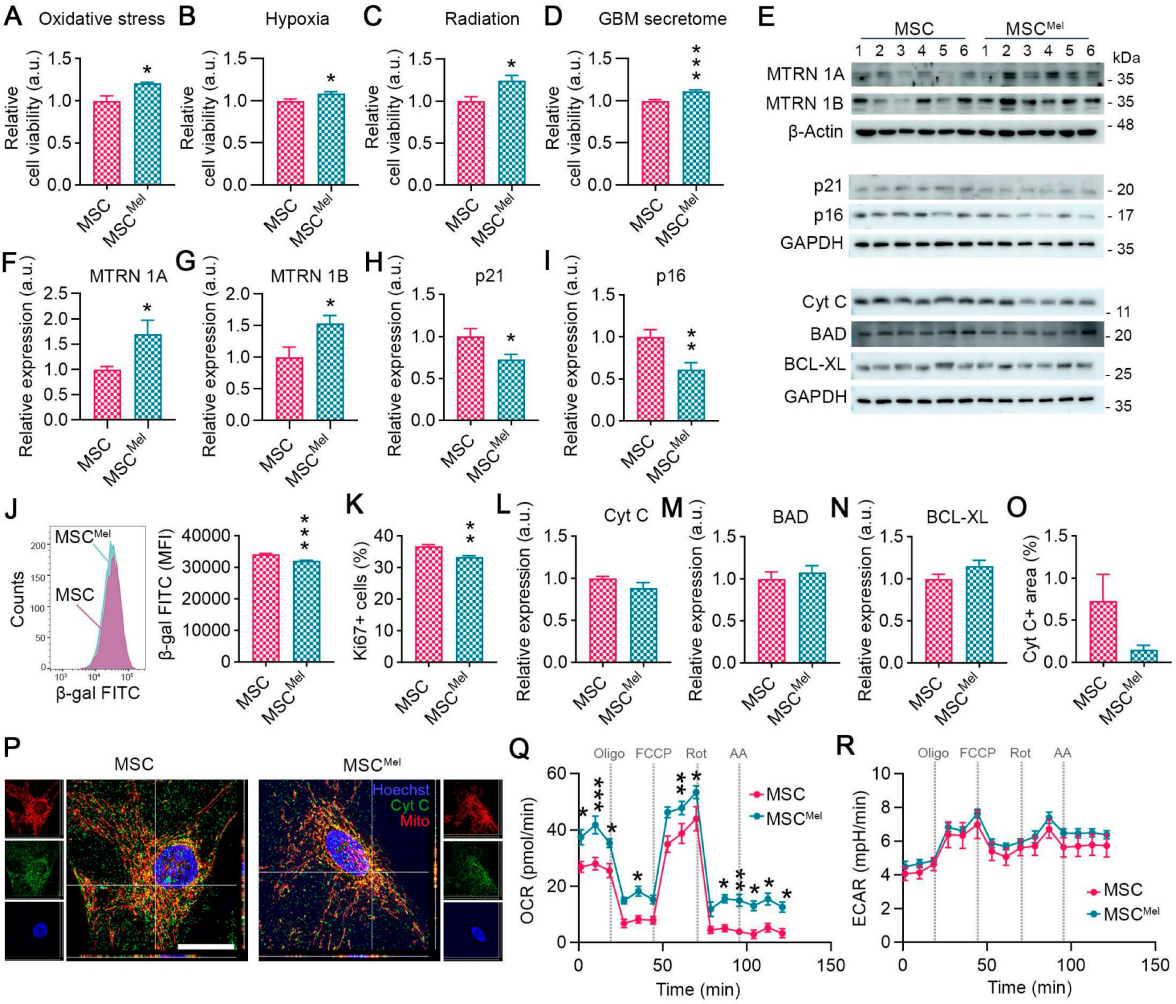
** Melatonin enhances the viability of human MSCs under tumor-like adverse conditions without altering their identity.** (**A**) Cell viability assay in MSC and MSC^Mel^ exposed to 1000 μM H_2_O_2_. Student's t test. (n = 4). (**B**) Cell viability assay in MSC and MSC^Mel^ exposed to 3% hypoxia. Student's t test. (n = 6). (**C**) Cell viability assay in MSC and MSC^Mel^ exposed to 25 Gγ radiation dose. Student's t test. (n = 6). (**D**) Cell viability assay in MSC and MSC^Mel^ exposed to GBM conditioned media. Student's t test. (n = 6). (**E**) Representative western blots of the different proteins analyzed in cultured MSCs and MSC^Mel^. (**F**) Densitometric quantification of MTRN1A western blot shown in panel E. Student's t test. (n = 6). (**G**) Densitometric quantification of MTRN1B western blot shown in panel E. Student's t test. (n = 6). (**H**) Densitometric quantification of p21 western blot shown in panel E. Student's t test. (n = 6). (**I**) Densitometric quantification of p16 western blot shown in panel E. Student's t test. (n = 6). (**J**) Senescence-associated β-galactosidase (β-gal) activity analyzed by flow cytometry. Student's t test. (n = 4). (**K**) Ki67+ proliferative cells analyzed by flow cytometry. Student's t test. (n = 5). (**L**) Densitometric quantification of Cyt C western blot shown in panel E. Student's t test. (n = 6). (**M**) Densitometric quantification of BAD western blot shown in panel E. Student's t test. (n = 6). (**N**) Densitometric quantification of BCL-XL western blot shown in panel E. Student's t test. (n = 6). (**O**) Immunofluorescence against Cyt C in cultured MSCs and MSC^Mel^. Student's t test. (n^MSC^ = 3, n^MSCMel^ = 4). (**P**) Representative immunofluorescence images of Cyt C (green) and MitoTracker (red) in cultured MSCs and MSC^Mel^. Scale bar: 25 µm. (**Q**) Oxygen consumption rate (OCR) on MSC and MSC^Mel^. Two-way ANOVA. (n = 5). (**R**) Extracellular acidification rate (ECAR) on MSC and MSC^Mel^. Two-way ANOVA. (n =5). Data are represented as mean ± SEM. *p < 0.05, **p < 0.01, ***p < 0.001 compared to control group (MSC).

Our initial step was to explore whether Mel pretreatment can enhance the resilience of MSCs under tumor-like adverse conditions. For this purpose, MSCs were pre-treated with 25 µm Mel for 24 h (MSC^Mel^). After verifying that Mel did not alter MSC identity (**Figure S1A-E**), cells were cultured under different hostile conditions, including oxidative stress, hypoxia, radiation and in the presence of the secretome of cancer cells. Mel pre-treatment increased cell viability of MSCs exposed to oxidative stress (1000 µM H_2_O_2_) or 3% hypoxia (**Figure 1A-B**). Similar results were observed when cellular stress was induced in MSCs by radiation (**Figure 1C**). Moreover, Mel pre-treatment was found to enhance cell viability of MSCs exposed to the secretome of GBM cells (**Figure 1D**). It is known that Mel exerts its beneficial effect by binding to its receptors MT1 (MTRN 1A) and MT2 (MTRN 1B), activating signaling pathways that govern main cellular processes, such as proliferation, senescence, apoptosis and metabolism. In our study, we found that Mel pre-treatment increased the levels of MTRN 1A and MTRN 1B in MSCs (**Figure 1E-G**). This was accompanied by a reduced expression of p21 and p16, which are involved in cell cycle arrest and senescence (**Figure 1E, H-I**). In addition, Mel treatment decreased the senescence-associated β-galactosidase activity in MSCs compared to untreated cells, even though it also reduced proliferation, as indicated by Ki67 expression (**Figure 1J-K** and **Figure S1F**). No differences were observed in the clonogenic capacity (**Figure S1G**). We then investigated the effects of Mel on apoptosis and found that the expression of apoptosis-regulatory proteins, such as Cyt C, BAD, and BCL-XL, remained unchanged (**Figure 1E, L-P**). Interestingly, Cyt C was less frequently located in the cytosol of MSC^Mel^, suggesting that Mel may prevent Cyt C release from mitochondria (**Figure 1O-P**) 30. In addition, we identified alterations in mitochondrial functional dynamics by measuring the oxygen consumption rate (OCR) (**Figure 1Q**). We observed elevated basal and maximal OCR in MSCs pre-treated with Mel, indicating enhanced mitochondrial function (**Figure 1Q**). Extracellular acidification rate (ECAR) in MSCs was unaltered by Mel, suggesting no effects in glycolysis (**Figure 1R**). In summary, these results suggest that Mel pre-treatment enhances MSC viability by improving cellular health, making the cells more resilient to a hostile environment.

### MSCs delay tumor growth in mouse glioblastoma models

**Figure 2:**
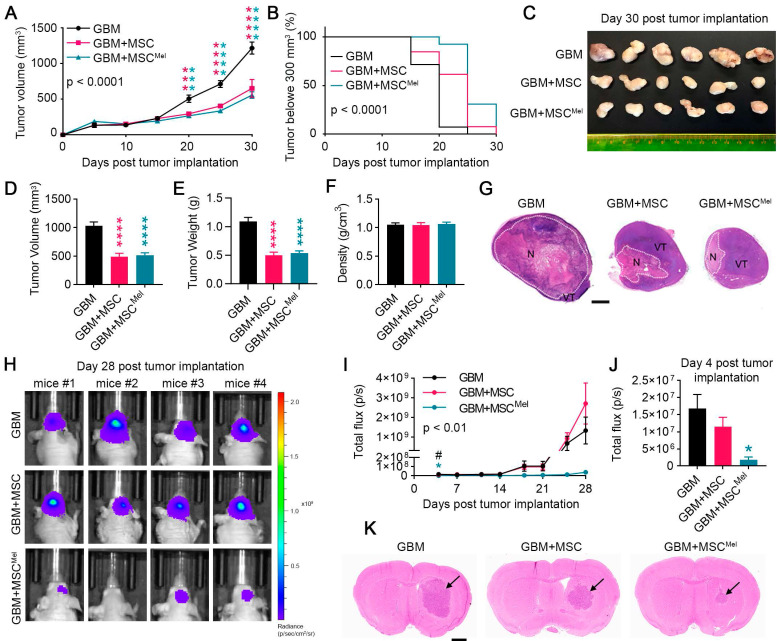
** MSCs delay tumor growth in mouse glioblastoma models.** Tumors were formed from GBM cells that were implanted in combination or not with MSCs or MSC^Mel^. (**A**) Tumor volume curves after subcutaneous injections of GBM, GBM+MSC, or GBM+MSC^Mel^ in mice. Statistically significant differences between groups at each time point are indicated in the graph (blue asterisk for GBM vs GBM+MSC and pink asterisk for GBM vs GBM+ MSC^Mel^). Two-way ANOVA. N^GBM^ = 14, N^GBM+MSC^ = 13, N^GBM+MSCMel^ = 13. (**B**) Kaplan-Meier plots of tumor growth in mice receiving subcutaneous injection of GBM, GBM+MSC, or GBM+MSC^Mel^. The statistical analysis for each comparison is as follow: GBM vs GBM+MSC p < 0.01, GBM vs GBM+MSC^Mel^ p < 0.0001, and GBM+MSC vs GBM+MSC^Mel^ p < 0.05. N^GBM^ = 14, N^GBM+MSC^ = 13, N^GBM+MSCMel^ = 13. (**C**) Representative images of tumors excised after sacrifice on day 30. Scale bar: 2 mm. (**D**) Tumor volume after resection on day 30. One-way ANOVA. N^GBM^ = 14, N^GBM+MSC^ = 13, N^GBM+MSCMel^ = 13. (**E**) Tumor weight after resection on day 30. One-way ANOVA. N^GBM^ = 14, N^GBM+MSC^ = 13, N^GBM+MSCMel^ = 13. (**F**) Tumor density after resection on day 30. One-way ANOVA N^GBM^ = 14, N^GBM+MSC^ = 13, N^GBM+MSCMel^ = 13. (**G**) Representative histological images of hematoxylin and eosin staining of the subcutaneous tumors showing the necrotic area (N) and viable area (VT). Note that GBM tumors show large necrotic areas. Scale bar: 2 mm. (**H**) Representative images showing in vivo bioluminescent signal in the head of mice at the end of the experiment (day 28 post intracranial tumor implantation). (**I**) Bioluminescent signal was quantified over time using the IVIS imaging system in total flux (photons/s). Statistically significant differences between groups at each time point are indicated in the graph (blue asterisk for GBM vs GBM+MSC, pink asterisk for GBM vs GBM+MSC^Mel^, hash symbol for GBM+MSC vs GBM+MSC^Mel^). Two-way ANOVA. N^GBM^ = 5, N^GBM+MSC^ = 5, N^GBM+MSCMel^ = 4. (**J**) Bioluminescent signals at day 4 post tumor implantation. One-way ANOVA. N^GBM^ = 5, N^GBM+MSC^ = 5, N^GBM+MSCMel^ = 4. (**K**) Representative histological images of hematoxylin and eosin-stained sections of the mouse brain, showing the developed tumors at day 28 post inoculation. Note that GBM+MSC^Mel^ mice show the smaller tumor. Scale bar: 1 mm. Data are represented as mean ± SEM. *p < 0.05, ***p < 0.001, ****p < 0.0001 compared to control group (GBM). ^#^p < 0.05 for the comparison GBM+MSC vs. GBM+MSC^Mel^.

Given that Mel enhanced the resilience of MSCs under tumor-like environment conditions, we decided to investigate how Mel pre-treatment affects the therapeutic properties of MSCs in mouse models of GBM. We used a cohort of mice to implant subcutaneous tumor xenografts 31. Animals were randomly assigned to three experimental groups: mice injected with U87 cells (GBM group), mice injected with U87 cells and MSCs (GBM+MSC group) and mice injected with U87 cells and MSCs pre-treated with Mel (GBM+MSC^Mel^ group). At 20 days after inoculation, GBM+MSC and GBM+MSC^Mel^ tumors exhibited a notable reduction in volume, as compared to the GBM group (**Figure 2A**). This effect persisted until the end of the study. Importantly, the combination of MSCs and Mel resulted in a greater proportion of tumors with a volume below 300 mm^3^, compared to the other groups (**Figure 2B**). While 92% of the tumors in the GBM+MSC^Mel^ groups were smaller than 300 mm^3^ by day 20, only 62% in the GBM+MSC groups and 7% in the GBM group maintained this reduced volume. At the study endpoint (i.e., 30 days post tumor implantation), volume and weight of resected tumors confirmed the delayed tumor growth in GBM+MSC and GBM+MSC^Mel^ groups, with no alterations in tumor density (**Figure 2C-F**). To analyze the histological features of the developed tumors, a subset of tissue samples was examined using hematoxylin and eosin staining. All tumors were characterized by two identifiable subregions: the viable area and the necrotic (hypoxic) region with inflammatory cell infiltration (**Figure 2G** and **Figure S2A**). While GBM tumors exhibited extensive necrosis, which correlates with a fast-growing phenotype, tumors with MSCs and MSC^Mel^ presented a predominant viable area. The presence of dying cells was further confirmed by the TUNEL staining (**Figure S2B-C**). When examining the viable tumor subregion, all tumors were highly cellular with frequent mitotic figures and vascularization, presenting common structural abnormalities of GBM, including atrophied blood vessels (**Figure S2A**). The proliferation index of the tumors was evaluated by Ki67 staining, which revealed a similar number of proliferative tumor cells in all groups (**Figure S2B-C**). In addition, the stem cell marker nestin identified foci enriched with nestin+ cells that were distributed throughout the tumor, irrespective of the experimental group. We did not find any significant difference in the percentage of area immunoreactive for nestin (**Figure S2B-C**). Interestingly, the number of CD34+ blood vessels was reduced in GBM+MSC tumors compared to control mice, suggesting that naïve MSCs exerted an anti-angiogenic effect (**Figure S2D**).

In order to model GBM in their natural microenvironment, another set of mice was used to perform an orthotopic xenograft tumor model. *In vivo* imaging revealed a significant reduction of bioluminescent signal in tumors inoculated with MSCs (pre-treated or not with Mel) as early as 4 days post-inoculation, with a more notable effect in the GBM+MSC^Mel^ group (**Figure 2H-J**). Importantly, tumors with MSC^Mel^ exhibited a sustained reduction in bioluminescent signal throughout the course of the experiment, compared to the control group, while naïve MSCs seemed to lose their effect at the end of the study (**Figure 2I**). However, this apparent loss of effect was likely an artifact, as evidenced by the histological analysis of the brains performed at the endpoint (i.e., 28 days post-tumor implantation). Consistent with the subcutaneous model, this analysis confirmed a delay in tumor growth in the presence of MSCs, and more notably in GBM+MSC^Mel^ mice (**Figure 2K**). No significant differences were observed in the expression of proliferation, stem cell and blood vessel markers between the groups (**Figure S2E-H**). In conclusion, the *in vivo* experimentation supported the notion that Mel pre-treatment enhanced the anti-cancer effect of MSCs in the early stage of tumor formation, thereby slowing GBM growth.

### Mel pre-treatment enhances the anti-migratory effects of MSCs in GBM cells

**Figure 3:**
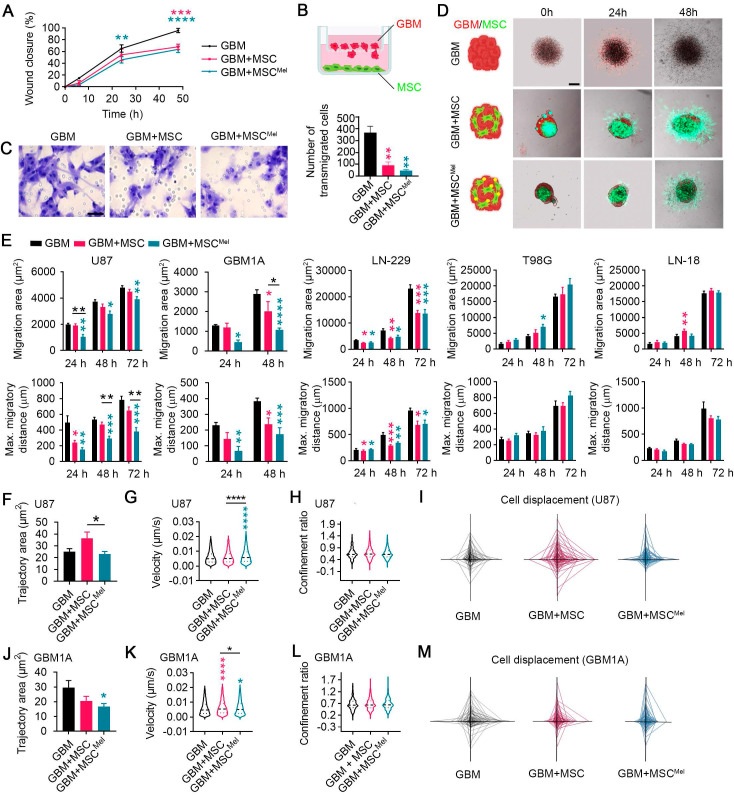
**Mel pre-treatment enhances the anti-migratory effects of MSCs in GBM cells.** Changes in the migratory pattern of GBM cells were determined by 2D and 3D *in vitro* assays. (**A**) Percentage of wound closure by GBM cells (U87) in a scratch assay. GBM cells were co-cultured with MSC or MSC^Mel^ at a 3:1 ratio. Statistically significant differences between groups at each time point are indicated in the graph (blue asterisk for GBM vs GBM+MSC and pink asterisk for GBM vs GBM+ MSC^Mel^). Two-way ANOVA. (n = 6 per group). (**B**) Schematic representation of the transwell assay. (**C**) Representative images of the transwell assay showing Nissl-stained transmigrated GBM cells (U87). Scale bar: 50 μm. The bar graph shows the number of GBM cells that have migrated through a transwell membrane after 48 h. One-way ANOVA. n = 3 per group. (**D**) Schematic and representative images of the tumorsphere migration model with GBM cells (CM-Dil, red) and MSCs (CMFDA, green). Scale bar: 100 μm. (**E**) Bar graphs depicting the migration area and maximum migratory distance of different GBM cell lines using a tumorsphere assay. One-way ANOVA. U87: N^GBM^ = 5, N^GBM+MSC^ = 4, N^GBM+MSCMel^ = 5; GBM1A: N^GBM^ = 5, N^GBM+MSC^ = 4, N^GBM+MSCMel^ = 5. LN-229: N^GBM^ = 6, N^GBM+MSC^ = 6, N^GBM+MSCMel^ = 6; T98G: N^GBM^ = 6, N^GBM+MSC^ = 6, N^GBM+MSCMel^ = 6; LN-18: N^GBM^ = 6, N^GBM+MSC^ = 6, N^GBM+MSCMel^ = 6. (**F**) Quantification of the trajectory area of U87 cells. One-way ANOVA. N^GBM^ = 291, N^GBM+MSC^ = 290, N^GBM+MSCMel^ = 252. (**G**) Violin plots depicting the instantaneous velocity in U87 cells. One-way ANOVA. N^GBM^ = 3659, N^GBM+MSC^ = 4451, N^GBM+MSCMel^ = 3486. (**H**) Violin plots depicting the confinement ratio in U87 cells. One-way ANOVA. N^GBM^ = 417, N^GBM+MSC^ = 517, N^GBM+MSCMel^ = 454. (**I**) Spider net graphs of U87 cell displacement. N^GBM^ = 43, N^GBM+MSC^ = 50, N^GBM+MSCMel^ = 50. (**J**) Quantification of the trajectory area of GBM1A cells. One-way ANOVA. (n = 150 cells per group). (**K**) Violin plots depicting the instantaneous velocity in GBM1A cells. One-way ANOVA. N^GBM^ = 4413, N^GBM+MSC^ = 5623, N^GBM+MSCMel^ = 4541. (**L**) Violin plots depicting the confinement ratio in GBM1A cells. One-way ANOVA. N^GBM^ = 486, N^GBM+MSC^ = 648, N^GBM+MSCMel^ = 486. (**M**) Spider graphs of GBM1A cells displacement (n = 50 per group). Data are represented as mean ± SEM. *p < 0.05, **p < 0.01, ***p < 0.001, ****p < 0.0001 compared to control group (GBM), unless otherwise indicated.

Proliferation, migration and invasion of cancer cells are key steps of tumor progression. Since the proliferative rate of tumor xenografts was similar among all experimental groups, we wanted to explore the ability of cancer cells to move when MSCs were present in the environment. For this purpose, we used 2D and 3D cell culture models, which allowed to better explore dynamic cell-cell interactions than *in vivo* models. To first interrogate the role of MSCs on cancer cell migration, we performed a scratch assay. GBM cells (i.e., U87 cells) showed a reduced collective migratory ability toward the scratched area when co-cultured with MSCs, and more markedly when MSCs were pre-treated with Mel (**Figure 3A** and **Figure S3A-B**). To discard any potential competence effect between GBM cells and MSCs, we used indirect co-culture methods to study migration. A transwell system was selected to culture GBM cells and MSCs or MSC^Mel^ preventing cell-cell contact (GBM cells were seeded in the upper chamber) (**Figure 3B**). Similar to direct co-cultures, GBM cells exhibited reduced ability to migrate through the transwell in the presence of MSCs, with a more pronounced effect observed in the GBM+MSC^Mel^ group (**Figure 3C**). Then, the capacity of MSCs to modulate the migration and invasion of GBM cells was assessed in 3D spheroids (**Figure 3D**). Consistent with the findings from 2D cultures, we observed that MSCs inhibit the migration of GBM cells (i.e., U87 cells) within spheroids (**Figure 3E**). In addition, MSCs pre-treated with Mel exhibited higher capacity to prevent GBM cell migration, compared to non-treated MSCs (**Figure 3E**). To validate this anti-migratory effect, we used other GBM cell lines with a different genetic background, including LN-229, T98G and LN-18 (**Figure S3C**), as well as primary GBM cells (i.e., GBM1A cells). Similar results to those observed in U87 tumorspheres were obtained with primary GBM1A cells, where MSCs pre-treated with melatonin exhibited a stronger inhibitory effect on migration (**Figure 3E**). In LN-229 tumorspheres, MSCs reduced tumor cell migration irrespective of melatonin pre-treatment, whereas no inhibitory effect was detected in T98G or LN-18 cells (**Figure 3E**). Then, cancer cell invasiveness was evaluated in spheroids seeded on matrigel. After 48 h, GBM cells (i.e., U87 cells) showed a decreasing trend to invade away from the sphere when MSC^Mel^ were present, but differences were not statistically significant (**Figure S3D-F**). Importantly, and in line with our *in vivo* data, the proliferative capacity of the GBM cells was not altered by MSCs or MSC^Mel^ in both 2D and 3D systems, thus ensuring a specific effect on migration (**Figure S3G-K**).

To further investigate deregulation of cell motility, we used single-cell tracking to measure the trajectory area of tumor cells in physical contact with MSCs. The value of this area is typically high for migratory cells and low for cells in which movement remains local 32. We found that the trajectory area of GBM cells (i.e., U87 cells) was decreased by Mel pre-treated MSCs, compared to that in the GBM+MSC group, while the velocity of GBM cells was increased (**Figure 3F-G**). To investigate directionality of cell movement, we analyzed the confinement ratio. While GBM cells showed different patterns of cell displacement between the groups, the confinement ratio remained unchanged (**Figure 3H-I**). A similar trend was observed when using primary GBM cells (i.e., GBM1A cells) (**Figure 3J-M**). Taken together, these data suggest that Mel promotes the close interaction of MSCs with GBM cells, impeding their dispersion. As a result, Mel enhances the anti-migratory effects of MSCs on GBM cells, yet it is important to note that this effect may vary depending on the inherent complexity and genotypic diversity of GBM subtypes.

### Identification of differentially expressed genes and pathways in GBM cells

**Figure 4:**
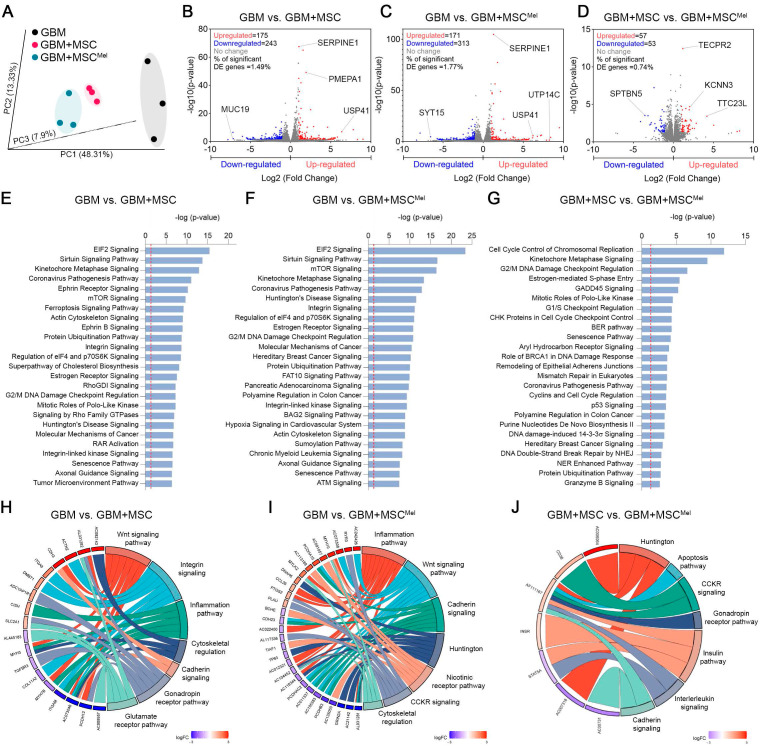
** Identification of differentially expressed genes and pathways in GBM cells.** RNA-seq analysis performed in the U87 cell line. (**A**) PCA of genes expressed in GBM, GBM+MSC and GBM+MSC^Mel^ comparisons. Plots represent individual samples (n = 3 per group). (**B**) Volcano plot showing the fold change and statistical significance of genes (p<0.05, log2 fold-change ≥ 1 or ≤ -1) expressed in the comparison GBM vs GBM+MSC (n = 3 per group). (**C**) Volcano plot showing the fold change and statistical significance of genes expressed in the comparison GBM vs GBM+MSC^Mel^ (n = 3 per group). (**D**) Volcano plot showing the fold change and statistical significance of genes expressed in the comparison GBM+MSC vs GBM+MSC^Mel^ (n = 3 per group). (**E**) Top modulated canonical pathways in the comparison GBM vs. GBM+MSC through IPA analysis (-log(p-value)>1.3). Reference line indicates threshold of significance (n = 3 per group). (**F**) Top modulated canonical pathways in the comparison GBM vs. GBM+MSC^Mel^ through IPA analysis (-log(p-value)>1.3). Reference line indicates threshold of significance (n = 3 per group). (**G**) Top canonical pathways in the comparison GBM+MSC vs. GBM+MSC^Mel^ through IPA analysis (-log(p-value)>1.3). Reference line indicates threshold of significance (n = 3 per group). (**H**) Chord plot showing association between exclusive genes differentially expressed in the comparison GBM vs. GBM+MSC (230 genes) and 7 first biological processes in which genes are implicated, using PANTHER. (**I**) Chord plot showing association between exclusive genes differentially expressed in the comparison GBM vs. GBM+MSC^Mel^ (275 genes) and 7 first biological processes in which genes are implicated, using PANTHER. (**J**) Chord plot showing association between exclusive genes differentially expressed in the comparison GBM+MSC vs. GBM+MSC^Mel^ (48 genes) and 7 first biological processes in which genes are implicated, using PANTHER.

Our next step was to explore the underlying molecular mechanisms of MSC-mediated tumor suppression using RNA sequencing (RNA-seq). Principal component analysis (PCA) showed that the transcriptional profile of the three experimental groups were robustly separated (**Figure 4A** and **Figure S4A**). The analysis of genes significantly modulated in the comparison GBM vs. GBM+MSC revealed 418 differentially expressed genes (DEG) (175 up-regulated and 243 down-regulated), while the comparison GBM vs. GBM+MSC^Mel^ showed 484 DEG (171 up-regulated and 313 down-regulated) (**Figure 4B-C**). Among these, the ubiquitin-specific protease 41 (USP41), a recently described deubiquitinase of Snail involved in migration of breast cancer cells 33, was among the most up-regulated transcripts in GBM cells exposed to MSCs or MSC^Mel^. Additionally, the comparison GBM+MSC vs. GBM+MSC^Mel^ evidenced that 110 genes were differentially expressed (57 up-regulated and 53 down-regulated) (**Figure 4D**). Of these, the down-regulation of the Spectrin Beta, Non-Erythrocytic 5 (*SPTBN5*) was particularly interesting, as this gene enables cytoskeletal protein binding activity, which is intimately associated with migration. The modulation of this gene in the comparison GBM+MSC vs. GBM+MSC^Mel^ suggests their potential contribution to the aforementioned improvements in the anti-migratory actions of MSC^Mel^ in GBM cells.

To gain further insight into the biological function of the DEG, we used IPA. We found modulations in core cellular processes involved in tumor progression, such as anabolism (ElF2 and mTOR signaling) and metabolic control (Sirtuin signaling), in GBM cells exposed to MSC or MSC^Mel^ (**Figure 4E-F**). Interestingly, several canonical pathways involved in cytoskeleton remodeling, including actin cytoskeleton, integrin, integrin linked kinase (ILK), and axonal guidance signaling, were significantly modulated in GBM cells exposed to MSC or MSC^Mel^ (**Figure 4E-F**). Additionally, IPA analysis revealed that among the top five molecular and cellular functions associated with all the genes in the three different comparisons, cellular movement and cellular assembly and organization were included (**Figure S4B**). Consistently, dysregulations of diseases and biological functions related to migration, invasion and movements of tumor cells were predicted in all comparations, with a more notable effect in the comparation GBM vs. GBM+MSC^Mel^ (**Figure S4C**).

Next, we analyzed the set of DEG specific for each comparison, which revealed specific alterations promoted by MSCs (230 genes in GBM vs. GBM+MSC) and MSC^Mel^ (275 genes GBM vs. GBM+MSC^Mel^) in GBM cells (**Figure S4D**). Notably, these specific genes were associated with alterations in cytoskeletal regulation, integrin signaling, cadherin signaling, and inflammation pathway in GBM cells exposed to MSCs or MSC^Mel^, according to PANTHER (Protein Analysis Through Evolutionary Relationships) knowledgebase (**Figure 4H-I**). Further analysis of the specific effects of Mel on the anti-cancer actions of MSCs (GBM+MSC vs. GBM+MSC^Mel^) revealed the presence of 48 specific genes that were differentially expressed (**Figure S4D**), which resulted in the alteration of pathways related to cadherin signaling, interleukin signaling, apoptosis, and Huntington (a pathway involved in inhibition of apoptosis via BDNF 34), among others (**Figure 4J**). These results reinforce the idea of the significant involvement of MSCs in regulating tumor cell behavior through cytoskeleton rearrangements and cell motility, which might be enhanced by Mel pre-treatment (**Figure S4E**).

### MSC^Mel^ influences cytoskeleton dynamics in GBM

**Figure 5:**
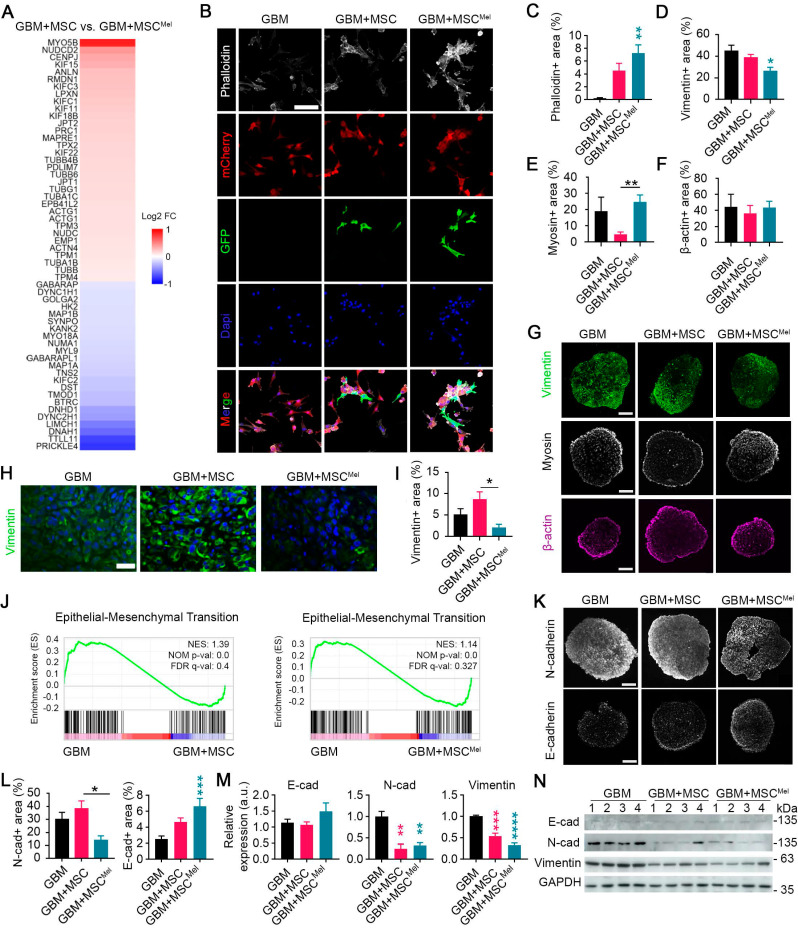
** MSC^Mel^ influences cytoskeleton dynamics in GBM.** (**A**) Relative abundance of remodeling cytoskeleton related significant genes (p-value<0.05) from the RNA-seq data in the comparison GBM+MSC vs. GBM+MSC^Mel^ (n = 3 per group) using PANTHER (U87 cell line). (**B**) Representative immunofluorescence images of F-actin marker (phalloidin) in GBM, GBM+MSC and GBM+MSC^Mel^ cultures using the U87 cell line. GBM cells were labeled with mCherry and MSCs were labeled with GFP. Scale bar: 100 μm. (**C**) Quantification of phalloidin+ area in the immunofluorescence shown in B. One-way ANOVA. (n = 3 per group). (**D**) Quantification of Vimentin+ area in U87 tumorspheres. One-way ANOVA. N^GBM^ = 8, N^GBM+MSC^ = 8, N^GBM+MSCMel^ = 6. (**E**) Quantification of β-actin+ area in U87 tumorspheres. One-way ANOVA. N^GBM^ = 5, N^GBM+MSC^ = 12, N^GBM+MSCMel^ = 12. (**F**) Quantification of myosin+ area in U87 tumorspheres. One-way ANOVA. N^GBM^ = 11, N^GBM+MSC^ = 10, N^GBM+MSCMel^ = 17. (**G**) Representative immunofluorescence images of U87 tumorspheres stained with vimentin (scale bar: 400μm) myosin (scale bar: 200 μm) and β-actin (scale bar: 200 μm). (**H**) Representative immunofluorescence images of vimentin staining in subcutaneous tumor xenografts. Scale bar: 50 μm. (**I**) Quantification of Vimentin+ area in the immunofluorescence shown in H. One-way ANOVA. N^GBM^ = 7, N^GBM+MSC^ = 5, N^GBM+MSCMel^ = 5. (**J**) Gene set enrichment analysis (GSEA) performed using RNA-seq data from GBM cells (U87). (**K**) Representative immunofluorescence images of U87 tumorspheres stained with N-cadherin (scale bar: 400 μm) and E-cadherin (scale bar: 200 μm). (**L**) Quantification of N-cadherin+ and E-cadherin+ area in the immunofluorescence shown in K. One-way ANOVA. N-cadherin N^GBM^ = 8, N^GBM+MSC^ = 7, N^GBM+MSCMel^ = 5. E-cadherin N^GBM^ = 17, N^GBM+MSC^ = 18, N^GBM+MSCMel^ = 18. (**M**) Densitometric quantification of N-cadherin, E-cadherin and Vimentin analyzed by western blotting in GBM cells (U87) exposed or not to MSC or MSC^Mel^ (n = 4 per group). (**N**) Representative western blots for the quantifications shown in M. Data are represented as mean ± SEM. *p < 0.05, **p < 0.01, ***p < 0.001, ****p < 0.0001 compared to control group (GBM), unless otherwise indicated.

The dynamic reorganization of the cytoskeleton is a crucial process in driving cell motility, ultimately influencing tumor progression. Therefore, we further focused on aspects involved in cytoskeleton dynamics. The transcriptomic analysis revealed that MSC^Mel^ altered a set of genes related to cytoskeleton remodeling in GBM cells, compared to the effects induced by untreated MSCs (**Figure 5A**). Among these genes, Myosin 5B (*MYO5B*) and NudC Domain Containing 2 (*NUDCD2*) appeared as the most up-regulated transcripts. Of these, changes in *MYO5B* were of particular interest, as previous studies have demonstrated that its inactivation promotes tumor cell invasion and motility 35,36. On the other hand, Prickle Planar Cell Polarity Protein 4 (*PRICKLE4*) and Tubulin Tyrosine Ligase Like 11 (*TTLL11*) were identified as the most downregulated genes in the comparison GBM+MSC vs GBM+MSC^Mel^. The downregulation of *TTLL11* was especially significant, since a recent report demonstrated that *TTLL11* regulates microtubule dynamics and is frequently altered in cancer 37.

To further investigate MSC^Mel^-induced cytoskeletal changes, we analyzed different cytoskeleton elements in GBM cells, using both *in vitro* and *in vivo* models. First, we performed phalloidin staining to examine tumoral expression of filamentous actin (F-actin), a major component of the cytoskeleton involved in migration and cell morphology (**Figure 5B**). A notable increased expression of F-actin was found in GBM cells (i.e., mCherry stained U87 cells) that were co-cultured with MSC^Mel^ in 2D system (**Figure 5B-C**). Similar results were found when using the primary GBM1A cell line (**Figure S5A-B**). Then, we decided to explore cytoskeletal remodeling in a physiologically relevant 3D environment by using U87 GBM tumorspheres (**Figure 5D-G**). A notable reduction in the expression of vimentin, the most common intermediate filaments in the cytoskeleton, was observed in GBM+MSC^Mel^ tumorspheres, compared to the GBM group (**Figure 5D, G**). In addition, the expression of myosin, a motor protein of the cytoskeleton, was reduced in GBM tumorspheres containing MSCs, compared to those with MSC^Mel^, while the expression of β-actin remained unaltered (**Figure 5E-G**). When shifting to the *in vivo* model, we found a decreased expression of vimentin in GBM+MSC^Mel^ xenografts, as compared to GBM+MSC tumors (**Figure 5H-I**). No significant changes were observed in the expression of β-actin, myosin and α-tubulin (a microtubule marker) in xenografts among the three groups of mice (**Figure S5C-D**). These results support the notion that Mel modulated the effects of MSCs in regulating the expression of genes involved in cell motility, thereby promoting the aforementioned enhanced anti-migratory effects of MSCs (**Figure 3**).

The alterations induced by MSC^Mel^ in vimentin expression prompted our suspicion regarding their involvement in the epithelial-mesenchymal transition (EMT) process, since vimentin is known to play a pivotal role in key events during EMT 38. By performing gene set enrichment analysis (GSEA), we further confirmed that the EMT pathway was significantly enriched in naïve GBM cells (i.e., U87 cells), compared to those exposed to MSC or MSC^Mel^ (**Figure 5J**). The hallmark of the EMT process is the loss of epithelial cell markers, such as E-cadherin, and the upregulation of mesenchymal markers, such as vimentin and N-cadherin. Consistent with the decreased expression of vimentin *in vitro* and *in vivo* (**Figure 5D, G-I**), we observed reduced expression of N-cadherin and increased expression of E-cadherin in GBM+MSC^Mel^ tumorspheres, compared to those in the GBM+MSC or GBM group, respectively (**Figure 5K-L**). A similar EMT profile was obtained by western blot analysis in GBM cells indirectly co-cultured with MSC or MSC^Mel^ (**Figure 5M-N**). In addition to the U87 cell line, we evaluated MSC^Mel^-induced changes in EMT-related proteins in T98G and LN-229 cells, which differ in their genetic backgrounds (**Figure S3C**). Across these GBM cell lines, MSC^Mel^ consistently induced a clear trend towards decreased vimentin expression, near statistical significance (*p*=0.05 for T98G and *p*=0.07 for LN-229), while the expression of N-cadherin and E-cadherin remained unaltered (**Figure S5E-H**). These results support the notion that vimentin is required for the EMT process, as suggested by previous research 39,40. Furthermore, they indicated that Mel pre-treated MSCs may reduce GBM aggressiveness by shifting the phenotype towards a less mesenchymal state, although the extent of this effect may vary depending on the biological complexity and heterogeneity inherent to each GBM subtype.

### MSC^Mel^ induced changes in extracellular matrix architecture of GBM

**Figure 6:**
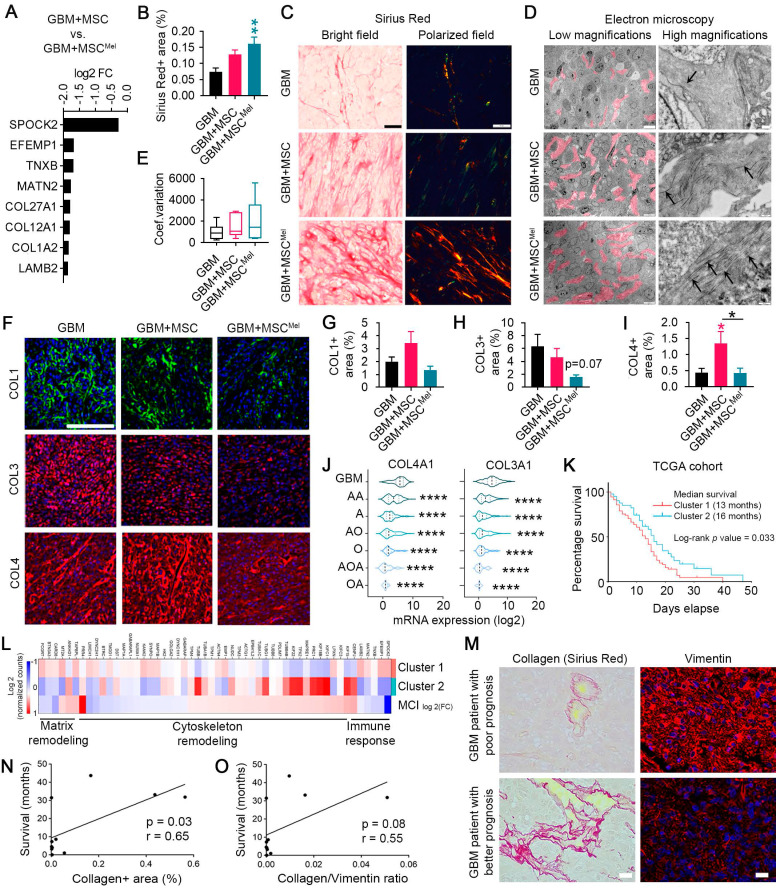
**MSC^Mel^ induced changes in ECM architecture of GBM.** (**A**) Relative abundance of significant genes (p-value<0.05) related to matrix remodeling from RNA-seq data in the comparison GBM+MSC vs. GBM+MSC^Mel^ using PANTHER. (**B**) Quantification of collagen fibers+ area stained with Sirius Red in subcutaneous tumor sections. Note the increased content of collagen in the GBM+MSC^Mel^ group. One-way ANOVA. N^GBM^ = 7, N^GBM+MSC^ = 7, N^GBM+MSCMel^ = 6. (**C**) Representative high magnification images of subcutaneous tumor sections stained with Sirius red, under bright field and polarized light microscopy. Scale bar: 50 μm. (**D**) Representative electron microscopy images of collagen fibers (pink color and arrows) in subcutaneous tumor sections. Scale bar: 10 µm (left) and 500 nm (right). (**E**) Coefficient of variation (CV) of the angle for all collagen fibers stained with Sirius Red in subcutaneous tumor sections. Note: Lowest CV correlates with the more aligned fibers. One-way ANOVA. N^GBM^ = 7, N^GBM+MSC^ = 7, N^GBM+MSCMel^ = 6. Whiskers indicate variability outside the upper and lower quartiles. (**F**) COL1, COL3 and COL4 immunofluorescence staining of subcutaneous tumor sections. Scale bar: 50 μm. (**G**) Quantification of COL1 (N^GBM^ = 6, N^GBM+MSC^ = 6, N^GBM+MSCMel^ = 5) in the immunofluorescence shown in F. One-way ANOVA. (**H**) Quantification of COL3 (N^GBM^ = 6, N^GBM+MSC^ = 5, N^GBM+MSCMel^ = 6) in the immunofluorescence shown in F. One-way ANOVA. (**I**) Quantification of COL4 (N^GBM^ = 7, N^GBM+MSC^ = 6, N^GBM+MSCMel^ = 6) in the immunofluorescence shown in F. One-way ANOVA. (**J**) mRNA expression (log2) of COL4A1 and COL3A1 in gliomas of different grades according to the TCGA database. (**K**) Survival curves of patient clusters according to TCGA database. Log-rank (Mantel-Cox) test (N^cluster 1^ = 96, N^cluster 2^ = 47). (**L**) Heat map of mRNA expression (Log2) of genes in the cluster 1 and cluster 2 and log2 fold-change for the comparison GBM+MSC vs. GBM+MSC^Mel^ using PANTHER. (**M**) Sirius Red and vimentin staining in GBM tissue samples from patients. Note that samples with low Sirius red staining frequently exhibited high vimentin expression, which tends to correlate with worse prognosis of patients. Scale bar: 20 µm. (**N**) Linear regression of progression-free survival of GBM patients and the collagen content in the tumor (% of collagen+ area). Pearson correlation. N = 11. (**O**) Linear regression of the progression-free survival of GBM patients and the ratio collagen/vimentin (% of collagen+ area / % of vimentin+ area). Pearson correlation. N = 11. A, astrocytoma; AA, anaplastic astrocytoma; AO, anaplastic oligodendroglioma; AOA, anaplastic oligoastrocytoma; O, oligodendroglioma; OA, oligoastrocytoma. Data are represented as mean ± SEM. *p < 0.05, **p < 0.01, ***p < 0.001, ****p < 0.0001 compared to control group (GBM), unless otherwise indicated.

Another important process for cell migration and invasion is extracellular matrix (ECM) remodeling. The ECM is composed of several molecules, including collagens, elastin and fibronectin, whose deposition or degradation can be modulated to facilitate tumor progression 41. Data from PANTHER pathways analysis of the DEG evidenced signaling pathways related to ECM remodeling, including those mediated by integrins and cadherins (**Figure 4H-J** and **Figure S4E**), which are important receptors for ECM components. In addition, we identified a list of genes involved in ECM remodeling that were downregulated in GBM cells exposed to MSC^Mel^, compared to GBM cells exposed to untreated MSCs (**Figure 6A**). Among these downregulated transcripts, we identified 3 collagen genes, including *COL1A2*, *COL12A1* and *COL27A1*. Interestingly, increased mRNA expression of *COL1A2* and *COL27A1* has been associated with poor survival in GBM patients, according to TCGA database (**Figure S6A**).

To better understand the biological significance of decreased collagen expression in GBM cells after MSC^Mel^ exposure, we evaluated the collagen content in the tumor xenografts by histopathological analysis. Increased deposition of packed collagen fibers was observed in Sirius Red stained sections of subcutaneous tumors with MSC^Mel^, compared to the GBM group (**Figure 6B-C**). Electron microscopy analysis confirmed this result by evidencing abundant collagen fibrils in the ECM of GBM+MSC^Mel^ tumors, while scarce, short fibrils were observed in the GBM group (**Figure 6D**). GBM+MSC tumors exhibited an intermediate collagen content at the ultrastructural level (**Figure 6D**). Confirmatory results of the increased collagen content in the GBM+MSC^Mel^ group were observed in orthotopic tumors, where a capsule-like structure was identified (**Figure S6B-C**). Arrangement of collagen fibers is an important process mediating tumor cell migration, being collagen alignment a predictor of malignancy for some tumors 42,43. Therefore, we analyzed collagen fiber orientation in tumor sections stained with Sirius Red (**Figure 6C, E**). Despite lack of statistical significance, tumor xenografts with MSC^Mel^ tended to have higher coefficient of variation of the angle for all collagen fibers (i.e., poor alignment) (**Figure 6E**), consistent with a less aggressive tumor phenotype 42,43. Next, we decided to study main collagen subtypes in GBM, including collagen type 1 (COL1), collagen type 3 (COL3) and collagen type 4 (COL4) (**Figure 6F-I**). While the expression of COL4 was increased in tumor xenografts with MSCs, compared to the GBM group, MSC^Mel^ prevented the accumulation of COL4 in the tumor environment (**Figure 6I**). No statistically significant differences were found in the expression of COL1 and COL3, although tumors with MSC^Mel^ exhibited a decreasing trend in COL3 content (**Figure 6G-H**). A similar expression pattern for COL1, COL3 and COL4 was observed *in vitro* using U87 tumorspheres, but only the effects on COL3 were statistically significant, with reduced COL3 expression in U87 tumorspheres treated with MSC^Mel^ (**Figure S6D-E**). We also analyzed the expression of COL3 in T98G and LN-229 cell lines, observing a significant decrease in LN-229 tumorspheres with MSC^Mel^ (**Figure S6F-G**). The reduction in the COL3 and COL4 induced by MSC^Mel^ in GBM tumorspheres was particularly interesting, as the mRNA expression levels of these collagens positively correlate with the malignancy grade of brain tumors in humans (**Figure 6J**). This positive correlation was also found for the mRNA expression level of *COL1* (**Figure S6H**). Altogether, these results sustain the concept that Mel pre-treatment modulates MSC-induced changes in ECM dynamics, shifting the tumor microenvironment towards a less pro-tumorigenic phenotype 44-47.

ECM components strongly influence the behavior of both normal and tumoral cells within the microenvironment. Among these cells, immune cells are known to play key roles in ECM homoeostasis by interacting directly with different ECM molecules 48. Given the well-known immunomodulatory properties of MSCs, we wondered whether changes in tumor ECM remodeling may be due to MSC-mediated inflammatory responses. As first evidence, the PANTHER pathway analysis of the DEG revealed activation of genes connected with the inflammation pathway and interleukin signaling (**Figure 4H-J** and **Figure S4E**).

To further evaluate how MSC and MSC^Mel^ influence the immunomodulatory phenotype in the tumor environment, we analyzed the cytokine profile in the supernatant of GBM cells co-cultured with MSC or MSC^Mel^ using indirect systems (i.e., without cell-cell contact). Overall, ELISA multiplexing evidenced that supernatants from indirect co-cultures of GBM and MSC^Mel^ exhibited a less inflammatory profile than that from the other groups (**Figure S6I**). Interestingly, we found opposite effects on the secretion of GM-CSF, G-CSF, INFγ, IL-6, IL-8, IL-9, MCP-1, VEGF, when comparing the supernatants from GBM+MSC and GBM+MSC^Mel^ (**Figure S6I**), suggesting that Mel pre-treated MSCs possess a more potent anti-inflammatory effect. Similar results were observed when using direct co-culture systems (i.e., with cell-cell contact) (**Figure S6J**). In addition, we examined the expression levels of the immunomodulatory cytokine GM-CSF in subcutaneous tumor sections. Immunofluorescence data indicated that the expression of GM-CSF was increased in subcutaneous tumors with naïve MSCs compared to the control group (i.e., GBM), while no statistically significant changes were found in tumors with MSC^Mel^ (**Figure S6K**). No differences were observed when examining the expression of the nuclear factor-κB (NF-κB), a mediator of the expression of inflammatory cytokines, including GM-CSF (**Figure S6K**). To further investigate changes in the immune environment, we analyzed the tumor-infiltrating leukocytes population in tumor xenografts, which is associated with increased survival in GBM patients 49-51. Immunostaining against CD45, a common leukocyte marker, revealed a more prominent presence of CD45+ tumor-infiltrating leukocytes in subcutaneous tumors grown with MSCs, compared to the GBM group (**Figure S6K**). This effect was potentiated by Mel pre-treated MSCs (**Figure S6K**). When examined the orthotopic tumor xenografts, we observed CD45+ cells within the tumors, as well as cells expressing the microglia marker Iba1, suggesting that immune cells also infiltrate the tumor in the brain (**Figure S6L**). Importantly, we observed that tumors with MSC^Mel^ are more prone to exhibit invading Iba1+ cells around the tumor (**Figure S6L**). Considered collectively, these findings suggest that Mel pre-treatment modulates the capacity of MSCs to influence the tumor immune environment, although further studies with fully immunocompetent mice are needed to extensively assess its impact on GBM progression.

### MSC^Mel^ provide a genetic fingerprint that predicts the survival of GBM patients

The RNA-seq analysis unveiled 110 DEG when comparing GBM+MSC vs. GBM+MSC^Mel^ (**Figure S4D** and **Figure S6M**). As we previously mentioned, many of these genes (i.e., 52 genes) were associated with matrix remodeling, cytoskeletal remodeling and immune response. Then, we selected these genes to generate a 52-gene signature, designated *MCI* (*M*atrix Remodeling, *C*ytoskeletal remodeling and *I*mmune response)*,* which was employed to categorize TCGA GBM patients based on RNA-seq data (**Figure S6N**). We identified two distinct clusters with variations in survival (median survival: 13 months for cluster 1 *vs* 16 months for cluster 2; p-value = 0.0329) (**Figure 6K**). Importantly, the gene expression profile identified in the comparison GBM+MSC vs. GBM+MSC^Mel^ shared genetic characteristics with cluster 2, which was associated with improved survival outcomes (**Figure 6K-L**).

In order to evaluate whether survival of GBM patients is associated with alterations in key components of the tumor environment, we performed immunohistochemical analyses in tumor samples from a cohort of 15 patients. Each sample was analyzed to determine the expression of collagen and vimentin, which frequently exhibited an opposite pattern (**Figure 6M**). Interestingly, a positive correlation was observed between collagen content and progression-free survival in GBM patients (**Figure 6N**). Finally, a nearly significant positive correlation between collagen/vimentin ratio and survival in GBM patients was observed (**Figure 6O**). These results support the idea that treatments targeting collagen and vimentin tumor content could potentially improve outcomes for GBM patients.

## Discussion

Despite numerous studies demonstrating the therapeutic effects of MSCs for different diseases, contradictory results have been reported when these cells are used for cancer treatment*.* While some studies indicate that MSCs have anticancer properties, others suggest that MSCs have protumorigenic actions 3. These discrepancies have raised safety concerns, limiting the application of MSCs in cancer patients. Our study provides evidence that melatonin exerts beneficial actions in enhancing the anti-cancer properties of MSCs, using both *in vitro* and *in vivo* models of GBM. To the best of our knowledge, this is the first study evaluating the combination of MSCs and Mel as a strategy intended to enhance the efficiency and safety of MSC-based therapies for cancer.

In this study, we demonstrate that Mel pre-treated MSCs delay tumor growth in orthotopic and subcutaneous GBM mouse models. Importantly, MSC^Mel^ produced remarkable effects in ECM dynamics, which is known to play an important role in cancer progression through changes in key components involved in tissues stiffness and cell-matrix interaction 41. In this context, MSC^Mel^ were found to increase tumor collagen deposition and to modulate molecules primarily associated with collagen folding, such as COL1A2 and COL12A1, suggesting a potential link between these molecular changes and collagen-mediated tumor growth inhibition. In line with these results, a previous report described that intratumoral administration of Mel induced the formation of a capsule of collagen in patient-derived xenografts of HNSCC 15. Retrospective studies have suggested that the formation of a fibrotic capsule could hinder the dissemination of tumor cells, thus improving cancer outcomes 52,53. It is important to highlight that migration is a key process in tumor progression, particularly at the tumor edge, where cells are more motile and invasive 54. By promoting the formation of a collagen capsule, MSC^Mel^ restrict the migration of cancer cells, potentially preventing tumor expansion and growth.

Consistent with all these findings, we found a positive correlation between collagen content and survival in GBM patients. Therefore, strategies aimed at increasing tumoral collagen may prevent tumor growth, as MSC^Mel^ treatment does. In this context, the organization of the collagen is of particular relevance. An elegant study has demonstrated that ECM remodeling resulting in enhanced collagen fiber alignment correlates with an escape from dormancy in human HNSCC and breast cancer models, thus promoting tumor growth 42. While aligned collagen fibers facilitate dispersion of tumor cells by creating migratory tracks, a random organization can hinder tumor invasion 42,55,56. In our study, we found that collagen-rich tumors exhibited reduced growth *in vivo*, which may respond to randomly oriented fibers. In addition, we demonstrated that GBM tumorspheres showed lower migratory capacity when MSC^Mel^ were present, in both primary and non-primary GBM cell lines. Consistently, we also found that MSC^Mel^ up-regulated MYO5B, an actin-based molecular motor whose inactivation promotes tumor cell invasion and motility 35,36, supporting a possible link to tumor growth inhibition. However, it is important to note that not all GBM cell lines consistently showed the same response to MSC^Mel^ treatment, suggesting that the GBM subtype may influence the effectiveness of this therapy. Despite this, these findings support the notion that MSC^Mel^ may remodel the ECM to prevent tumor progression, thus exerting anti-tumor effects. Further investigations should be conducted to fully determine whether these architectural rearrangements induced by MSC^Mel^ result in a low degree of linear collagen fiber orientation, potentially driving cancer dormancy, as supported by previous evidence 42.

A recent study demonstrated that collagen remodeling is regulated by vimentin 57. In particular, vimentin was found to associate with myosin 10 to increase collagen degradation in a membrane-type 1 matrix metalloproteinase (MT1-MMP)-dependent manner. This study is consistent with our findings, indicating that the presence of MSC^Mel^ leads to tumors exhibiting reduced expression of vimentin and high collagen content, suggesting that defects on vimentin contribute to collagen accumulation. In addition, it is well established that vimentin plays a crucial role in regulating EMT, a pivotal process in tumor progression 58. In experimental models of colon cancer, the downregulation of vimentin expression by miRNA-17-5p was found to inhibit EMT and metastasis 59,60. These observations are consistent with our results indicating that Mel pre-treated MSCs may be involved in the activation of transcriptional programs that prevent vimentin expression and EMT. However, opposing reports have indicated that MSCs stimulate EMT 61-63. For instance, previous studies using direct co-cultures of breast or gastric cancer cells with bone marrow-derived MSCs identified an upregulation of EMT markers, including vimentin, N-cadherin, Twist, and Snail, while observed reduced expression of E-cadherin 61,62. Another study showed that bone marrow-derived MSCs enhance de novo production of lysyl oxidase in breast carcinoma cells, thus promoting Twist-induced EMT and increasing metastasis 44. The discrepancies in the ability of MSCs to promote or suppress EMT may be attributable to differences in the experimental design (e.g., direct vs indirect co-cultures), tumor type, tumor microenvironments, MSC source, or proportion of MSCs in the tumor, among other factors. We speculate that treating MSCs with Mel might enhance their proportion within the tumor during the early stages of growth, which may favor a more efficiently modulation of critical tumor processes, such as EMT. This hypothesis arises from our findings showing that melatonin makes MSCs more resilient to tumor-like adverse conditions, including oxidative stress and hypoxia, among others. Although MSC^Mel^ are expected to gradually disappear over time, as suggested by other studies 64-66, the early suppression of EMT may account for lasting effects that continue to influence tumor progression even in their absence. Our study underscores the importance of intracellular and extracellular reorganizations in tumor development.

Evidence suggests that, similar to the collagen-rich matrix impeding tumor cell dispersion 52,53, collagen barriers may also hinder the infiltration of immune cells into the tumor 67,68. In that case, the immune-excluded tumor phenotype induced by collagen content would promote tumor growth 69. However, we found a positive correlation between collagen deposition and the presence of tumor-infiltrating immune cells, such as leukocytes and microglia, that was associated with decreased tumor size. This may indicate that collagen remodeling mediated by MSC^Mel^ prevents the migration of cancer cells from the tumor, while not impeding the infiltration of immune cells. This is a very relevant observation since the presence of immune cells has been positively associated with survival of GBM patients 49-51. In line with this, a recent study highlighted the critical role of microglia in promoting anti-tumor immunity and suppressing brain metastasis 70. Despite the increased number of tumor-infiltrating immune cells, we found that experimental models of GBM containing MSC^Mel^ exhibited an anti-inflammatory phenotype. A possible explanation for this fact could be that the anti-inflammatory milieu generated by MSCs activates compensatory mechanisms favoring the infiltration of immune cells. A similar phenomenon has been found in sepsis and traumatic brain injury models, in which the organism reacts to the insult with a severe inflammatory response and, at the same time, activates mechanisms to counteract the inflammation 71,72. It is also important to note that each tumor microenvironment induce a particular inflammatory response in MSCs that may influence their capacity to hinder tumor growth. For instance, MSCs are known to secrete different inflammatory factors, the most important being IFNγ, TNFα and IL-1β 73. These cytokines can act as pro-inflammatory or anti-inflammatory factors depending on the local conditions. In our study, we found that the environment of GBM cells exposed to MSC^Mel^ presented reduced levels of IFNγ, TNFα and IL-1β, and this condition was associated with a less aggressive tumor phenotype. Collectively, these findings suggest that the diverse immunomodulatory actions of MSCs ultimately contribute to reduce tumor immune escape, addressing the concerns raised by studies that have questioned MSC application for cancer 74-76.

Although the clinical use of MSC in cancer has been limited due to safety concerns, many ongoing studies are using MSCs as Trojan horses to deliver drugs into the tumor, relying on the tumor-tropic property of MSCs 77-80. This includes clinical studies investigating the safety and efficacy of MSC engineered to express antitumor agents in patients with advanced colorectal cancer and metastatic solid tumors (NCT06446050 and NCT05699811). In this context, it would be interesting to explore whether engineering MSCs to release higher levels of melatonin might potentiate their anti-tumor effects in future studies. Previous research suggests that modulating the expression of enzymes involved in Mel synthesis from tryptophan or altering the levels of melatonin transporters 81,82, could lead to more effective Mel secretion. These approaches would not only take advantage of the inherent therapeutic actions of MSCs, but also the benefits derived from the oncostatic properties of Mel 15-20. By the moment, our work demonstrates that Mel pre-treatment potentiates the anti-tumor actions of MSCs in experimental GBM models. In particular, we found a retarded tumor growth that was associated with increased collagen deposition, reduced vimentin expression and inhibited migration of GBM cells in the presence of MSC^Mel^. These results underscore the promise of using cell engineering techniques to convert MSCs into Trojan horses secreting high levels of melatonin to offer innovative therapeutic strategies in GBM patients.

Finally, it is essential to recognize that GBM is a highly heterogeneous disease, with distinct subtypes characterized by different molecular and cellular profiles that can influence therapeutic responses. Thus, the effects of MSC^Mel^ may vary depending on the biological complexity and specific genotypic characteristics of each GBM subtype. Future studies that fully address these variables are necessary to better understand the broader applicability and limitations of MSC^Mel^ therapy. Additionally, while we did not include survival analyses in our current study, incorporating them in future research could provide a more comprehensive evaluation of the long-term impact of MSC^Mel^ in brain tumor models. Despite these considerations, our data highlight a novel role of Mel in modulating the anti-cancer properties of MSCs. This study provides a basis for the use of Mel to improve the safety and efficacy of MSC-based therapies, paving the way for clinical trials combining melatonin and MSCs for GBM patients.

## Supplementary Material

Supplementary figures and table.
