# Supplementary material for: Glioblastoma progression is hindered by melatonin-primed mesenchymal stromal cells through dynamic intracellular and extracellular reorganizations
Source: Theranostics. 2025 Feb 10;15(7):3076–97. doi: 10.7150/thno.104143 (PMC11898303; doi:10.7150/thno.104143)
Supplement: Supplementary file 1 — Supplementary figures and table. [file thnov15p3076s1.pdf]

Glioblastoma progression is hindered by melatonin-primed mesenchymal stromal cells through dynamic intracellular and extracellular reorganizations

Laura Olmedo-Moreno, Concepción Panadero-Morón, Jesús María Sierra-Párraga, Rubén Bueno-Fernández, Emily S. Norton, Yolanda Aguilera, Nuria Mellado-Damas, Patricia García-Tárraga, Raquel Morales-Gallel, María Antequera-Martínez, Raúl V. Durán, Jaime Ferrer-Lozano, Germaine Escames, José Manuel García-Verdugo, Alejandro Martin-Montalvo, Hugo Guerrero-Cázares, Vivian Capilla-González✉

Supplementary data

FIGURES AND TABLES

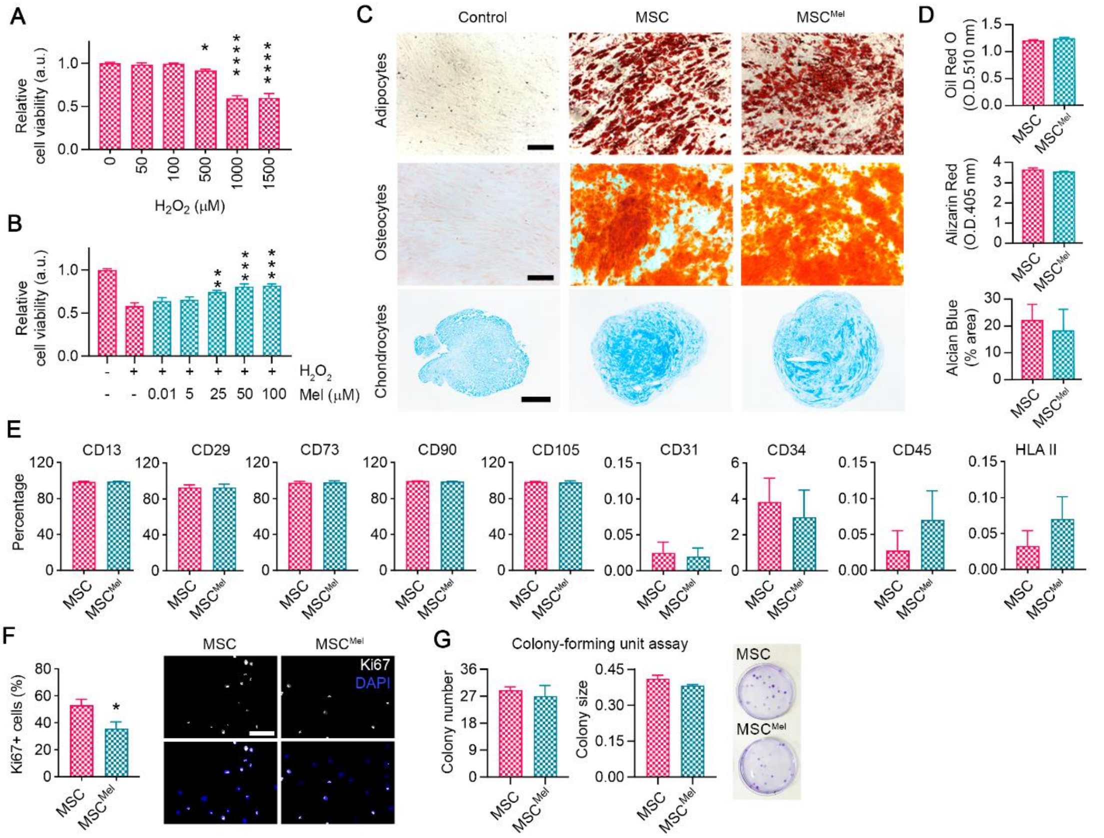

Figure S1. Melatonin enhances the viability of human MSCs under tumor-like adverse conditions without altering their identity. (**A**) Optimization of the cytotoxic effect of increasing concentrations of H_2O_2 (0–1500 μM), measured by Alamar Blue viability assay. One-way ANOVA. n = 3 per group. * $p < 0.1$, **** $p < 0.0001$ compared to control group (0 μM). (**B**) Viability measurement of MSCs and MSC^{Mel} exposed to 1000 μM H_2O_2, by Alamar Blue viability assay. One-way ANOVA. n = 4 per group. ** $p < 0.01$, *** $p < 0.001$ compared to control group (0 μM Mel plus 1000 μM H_2O_2). (**C**) Representative optical images showing the morphological aspect of MSCs differentiated into adipocytes (Oil Red O; scale bar: 200 μm), osteocytes (Alizarin Red; scale bar: 200 μm) and chondrocytes (Alcian Blue; scale bar: 100 μm). (**D**) Quantification of differentiation stainings shown in C. Student's t-test. n = 3 per group. (**E**) Flow cytometry analysis of MSC and MSC^{Mel} showing positive staining for CD13, CD29, CD73, CD90 and CD105, and negative staining for CD31, CD34, CD45 and HLA-II surface markers. Student's t-test. n = 4 per group. (**F**) Immunofluorescence against Ki67 in cultured MSCs and MSC^{Mel}. Student's t-test. n = 4 per group. *$p < 0.05$ compared to control group. Scale bar: 100 μm. (**G**) Colony-forming assay performed in MSCs and MSC^{Mel} cultured for 14 days. The number and size of the colonies was counted after staining with cresyl violet. Student's t-test. n = 3 per group. Data are represented as mean ± SEM.

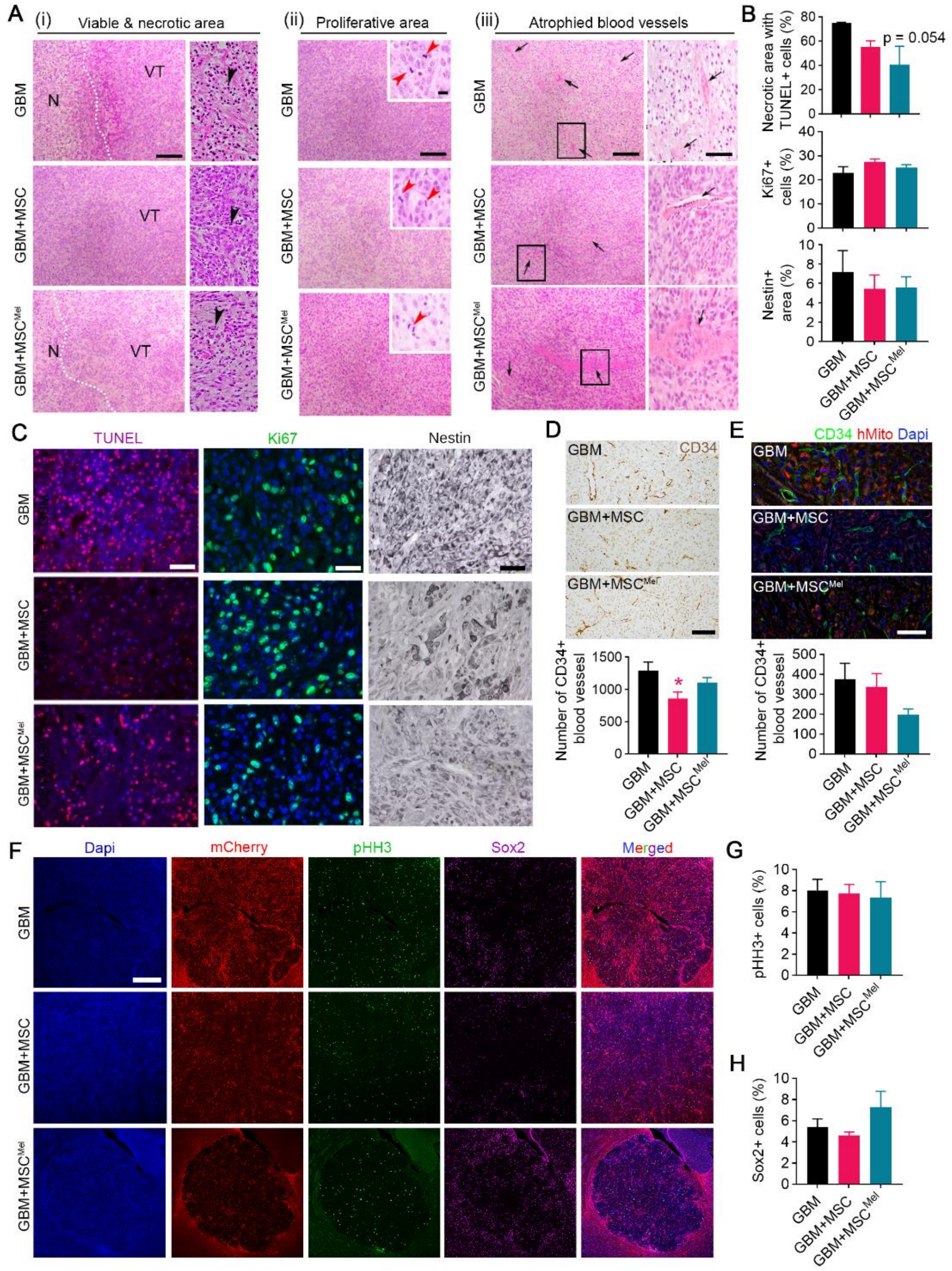

Figure S2. MSCs delay tumor growth in mouse glioblastoma models. (**A**) Representative histological images of hematoxylin and eosin-stained sections of the subcutaneous tumors. Panel (i), left, shows images of the necrotic area (N) and viable area (VT). Note that mice inoculated with GBM cells alone show large necrotic areas. Scale bar: 200 µm. Panel (i), right, shows a detail of the immune infiltrates (arrows). Panel (ii) shows the proliferative areas with mitotic cells (red arrowhead in inserts). Scale bar: 20 µm (200 µm for insert). Panel (iii), shows atrophied blood vessels (black arrow). Scale bar: 50 µm (200 µm for insert). (**B**) Quantification of the necrotic area in subcutaneous tumor sections measured as the percentage of area containing TUNEL+ cells. One-way ANOVA. $N^{GBM} = 4$, $N^{GBM+MSC} = 5$, $N^{GBM+MSCMel} = 4$. Quantification of the percentage of Ki67+ cells in subcutaneous tumor sections. One-way ANOVA. $N^{GBM} = 7$, $N^{GBM+MSC} = 7$, $N^{GBM+MSCMel} = 5$. Quantification of Nestin+ area in subcutaneous tumor sections. One-way ANOVA. $N^{GBM} = 6$, $N^{GBM+MSC} = 4$, $N^{GBM+MSCMel} = 6$. (**C**) Representative images of immunostaining for TUNEL, Ki67 and nestin in the subcutaneous tumor sections. Scale bar: 50 µm. (**D**) Immunostaining for CD34 in subcutaneous tumor sections. Scale bar: 100 µm. One-way ANOVA. $N^{GBM} = 6$, $N^{GBM+MSC} = 7$, $N^{GBM+MSCMel} = 6$.

(**E**) Immunostaining for CD34 and human mitochondria (hMito) in orthotopic tumor sections. Scale bar: 50 µm. One-way ANOVA. $N^{GBM} = 5$, $N^{GBM+MSC} = 5$, $N^{GBM+MSCMel} = 4$. (**F**) Immunostaning for m-Cherry, pHH3 and Sox2 in orthotopic tumor sections. Scale bar: 500 µm. (**G**) Quantification of the percentage of pHH3+ cells in orthotopic tumor sections. One-way ANOVA. $N^{GBM} = 5$, $N^{GBM+MSC} = 5$, $N^{GBM+MSCMel} = 4$. (**H**) Quantification of Sox2+ area in orthotopic tumor sections. One-way ANOVA. $N^{GBM} = 5$, $N^{GBM+MSC} = 5$, $N^{GBM+MSCMel} = 4$. Data are represented as mean ± SEM. *$p < 0.05$ compared to control group (GBM).

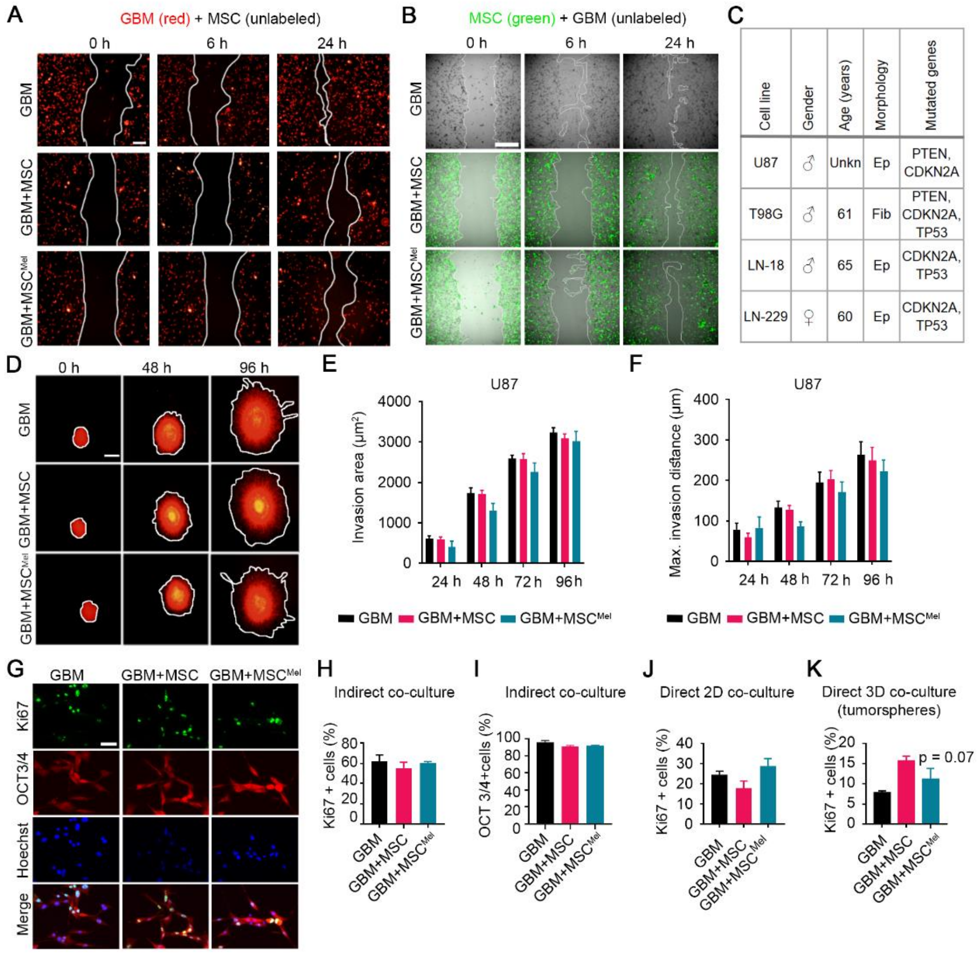

Figure S3. Mel pre-treatment enhances the anti-migratory effects of MSCs in GBM cells. (**A**) Representative images of the wound scratch assay using GBM (U87) and MSC co-cultures. GBM cells were stained with CM-DiI dye for visualization. Scale bar: 200 μm. (**B**) Representative images of the wound scratch assay using GBM (U87) and MSC co-cultures. MSCs were stained with CMFDA dye for visualization. Scale bar: 500 μm. (**C**) Characteristics of the commercial cell lines used in this study. Ep: Epithelial. Fib: Fibroblast. Unkn: Unknown. (**D**) Representative images of the U87 tumorspheres at 0, 48 and 96 h after seeding, showing the invasion of U87 cells (red). Scale bar: 200 μm. (**E**) Bar graph depicting the invasion area in the assay shown in D. One-way ANOVA. n = 5 per group. (**F**) Bar graph depicting the maximum invasion distance in the assay shown in D. One-way ANOVA. n = 5 per group. (**G**) Representative immunofluorescence images of GBM cells (U87) indirectly co-cultured with MSC or MSC^{Mel}. The assessed markers were Ki67 (proliferation) and OCT3/4 (stemness). (**H**) Bar graphs depicting the percentage of Ki67+ cells in an immunofluorescence assay. One-way ANOVA. $N^{GBM} = 4$, $N^{GBM+MSC} = 4$, $N^{GBM+MSCMel} = 3$. (**I**) Bar graphs depicting the percentage of OCT3/4+ cells in an immunofluorescence assay. One-way ANOVA. n = 3 per group. (**J**) Bar graphs depicting the percentage of Ki67+ GBM cells (U87) co-cultured with MSCs in a direct 2D system, using flow cytometry. One-way ANOVA. n = 7 per group. (**K**) Bar graphs depicting the percentage of Ki67+ GBM cells (U87) co-cultured with MSCs in a direct 3D system (tumorspheres), using flow cytometry. One-way ANOVA. $N^{GBM} = 3$, $N^{GBM+MSC} = 3$, $N^{GBM+MSCMel} = 4$. Data are represented as mean ± SEM. $^{**}p < 0.01$, $^{***}p < 0.001$, $^{****}p < 0.0001$ compared to control group (GBM).

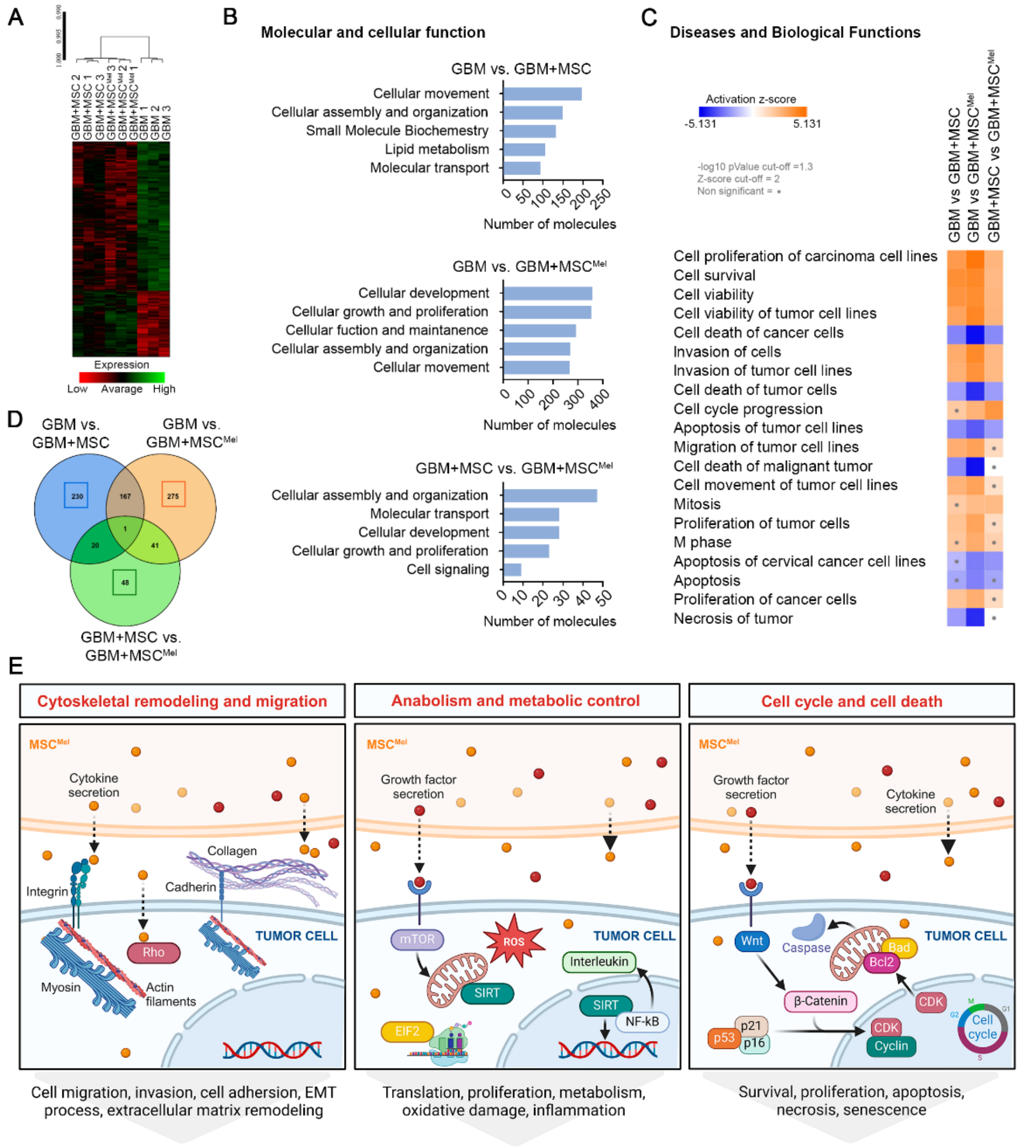

Figure S4. Identification of differentially expressed genes and pathways in GBM cells. RNA-seq analysis performed in the U87 cell line. (**A**) Heat map of genes analyzed by Illumina, grouped into clusters based on expression profiles (n = 3 per group). (**B**) Molecular and cellular functions category predicted by IPA analysis for each comparison. (**C**) Diseases and biological functions predicted to be modulated in each comparison using IPA. Z-score was used to predict activation (Z-score ≥ 2; orange) or inhibition (Z-score ≤ − 2; blue) (n = 3 per group). (**D**) Venn diagram showing the common and specific genes in the three comparisons (n = 3 per group). (**E**) Summary figure of the key pathways affected by MSC^{Mel} in the tumor cells according to RNA-seq data. The summary figure was created with BioRender.

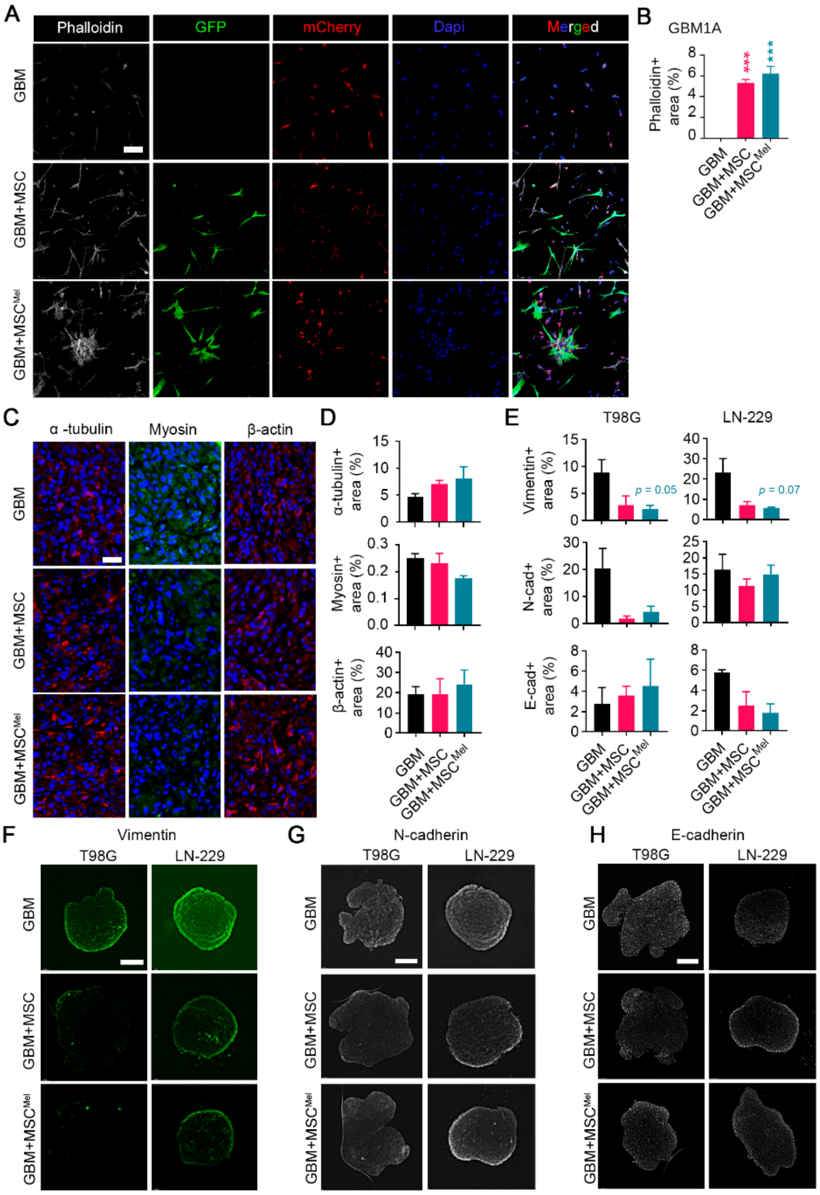

Figure S5. MSC^{Mel} influences cytoskeleton dynamics in GBM. (**A**) Representative immunofluorescence images of F-actin marker (phalloidin) in GBM, GBM+MSC and GBM+MSC^{Mel} cultures using the GBM1A cell line. GBM cells were labeled with m-Cherry and MSCs were labeled with GFP. Scale bar: 200 μm. (**B**) Quantification of phalloidin+ area in the immunofluorescence shown in A (n = 3 per group) (**C**) Representative immunofluorescence images of α-tubulin, myosin and β-actin in subcutaneous tumor xenografts. Scale bar: 25 μm. (**D**) Quantification of the expression of α-tubulin (N^{GBM} = 7, $N^{GBM+MSC}$ = 6, $N^{GBM+MSCMel}$ = 4), myosin (N^{GBM} = 7, $N^{GBM+MSC}$ = 7, $N^{GBM+MSCMel}$ = 4) and β-actin (N^{GBM} = 7, $N^{GBM+MSC}$ = 7, $N^{GBM+MSCMel}$ = 4) in the immunofluorescence assay shown in C. One-way ANOVA. (**E**) Immunostaining analysis of EMT-related proteins in T98G and LN-229 tumorspheres. Vimentin in T98G: N^{GBM} = 7, $N^{GBM+MSC}$ = 4, $N^{GBM+MSCMel}$ = 6. Vimentin in LN-229: N^{GBM} = 7, $N^{GBM+MSC}$ = 5, $N^{GBM+MSCMel}$ = 5. N-cadherin in T98G: N^{GBM} = 7, $N^{GBM+MSC}$ = 4, $N^{GBM+MSCMel}$ = 6. N-cadherin in LN-229: N^{GBM} = 7, $N^{GBM+MSC}$ = 5, $N^{GBM+MSCMel}$ = 5. E-cadherin in T98G: N^{GBM} = 8, $N^{GBM+MSC}$ = 7, $N^{GBM+MSCMel}$ = 4. E-cadherin in LN-229: N^{GBM} = 3, $N^{GBM+MSC}$ = 5, $N^{GBM+MSCMel}$ = 6. One-way ANOVA. (**F**) Representative immunofluorescence images of vimentin in T98G and LN-229 tumorspheres. Scale bar: 250 μm. (**G**) Representative immunofluorescence images of N-cadherin in T98G and LN-229 tumorspheres. Scale bar: 250 μm. (**H**) Representative immunofluorescence images

of E-cadherin in T98G and LN-229 tumorspheres. Scale bar: 250 μm. Data are represented as mean ± SEM. ***p < 0.001 compared to control group (GBM).

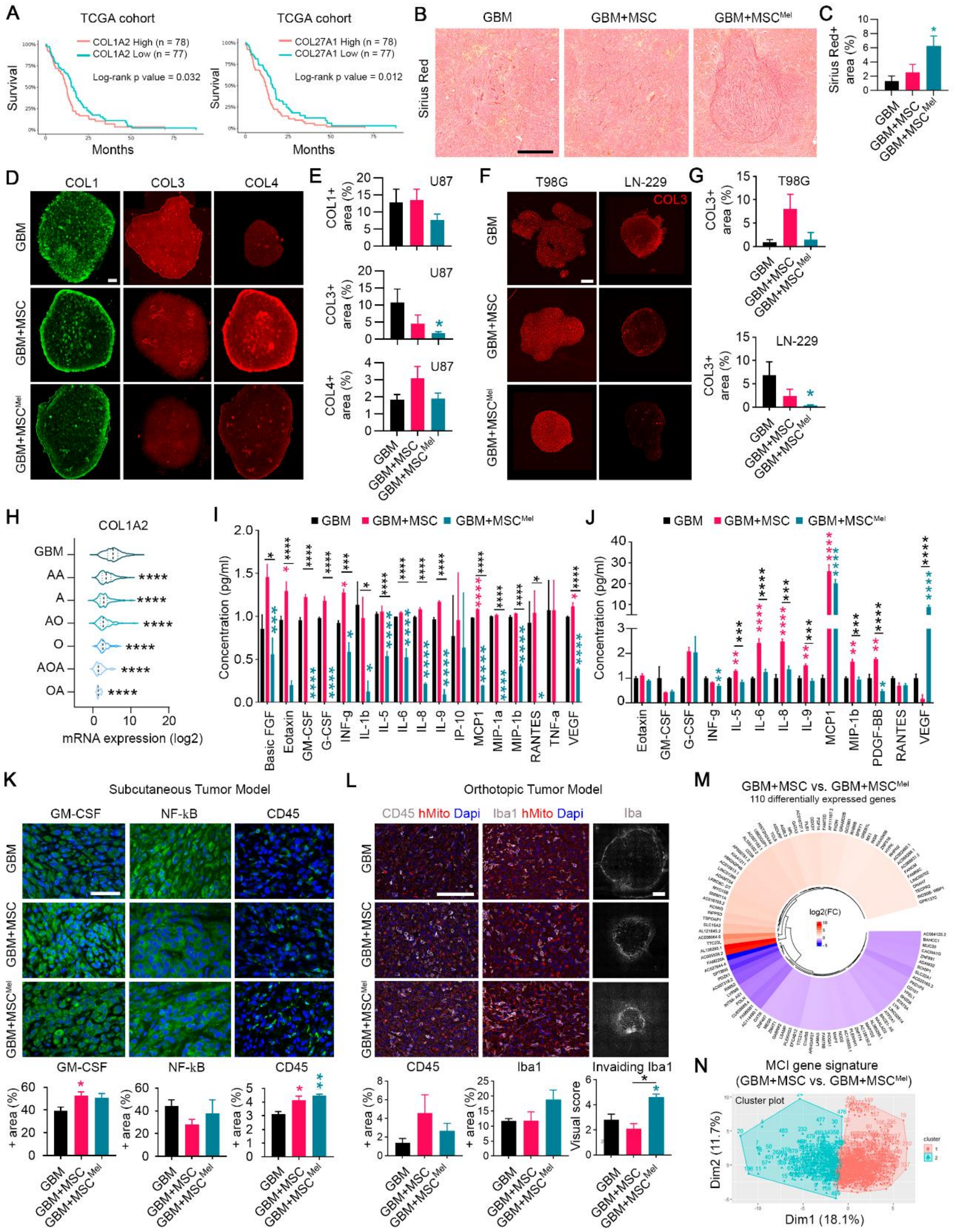

Figure S6. MSC^{Mel} induced changes in ECM architecture of GBM. (**A**) Survival curve of patients with high and low expression of *COL1A2* or *COL27A1* according to the TCGA database. (**B**) Sirius Red staining in orthotopic brain tumor sections. Scale bar 300 µm. (**C**) Quantification of the area stained with Sirius Red in the orthotopic brain tumor sections. (**D**) COL1, COL3 and COL4 immunofluorescence staining in the U87 tumorsphere assay. Scale bar: 200 µm. (**E**) Quantification of COL1 (N^{GBM} = 12, $N^{GBM+MSC}$ = 9, $N^{GBM+MSCMel}$ = 13), COL3 (N^{GBM} = 10, $N^{GBM+MSC}$ = 9, $N^{GBM+MSCMel}$ = 16) and COL4 (N^{GBM} = 12, $N^{GBM+MSC}$ = 9, $N^{GBM+MSCMel}$ = 12) in the immunofluorescence shown in D. One-way ANOVA. (**F**) COL3 immunofluorescence staining in tumorsphere assays using T98G and LN-229 cells. Scale bar: 200 µm. (**G**) Quantification of COL3 in the immunofluorescence shown in F. T98G: N^{GBM} = 7, $N^{GBM+MSC}$ = 7, $N^{GBM+MSCMel}$ = 3. LN-229: N^{GBM} = 4, $N^{GBM+MSC}$ =

5, $N^{GBM+MSCMel}$ = 6. One-way ANOVA. (**H**) mRNA expression (log2) of *COL1A2*. (**I**) Quantification of cytokines levels (pg/ml) secreted in the media of indirect co-cultures. (**J**) Quantification of cytokines levels (pg/ml) secreted in the media of direct co-cultures. (**K**) GM-CSF, NF-KB and CD45 immunofluorescence staining of subcutaneous tumor sections. Scale bar: 50 µm. Quantification of GM-CSF+ area (N^{GBM} = 7, $N^{GBM+MSC}$ = 7, $N^{GBM+MSCMel}$ = 6). Quantification of NF-KB+ (N^{GBM} = 7, $N^{GBM+MSC}$ = 6, $N^{GBM+MSCMel}$ = 6). Quantification of CD45+ area (N^{GBM} = 6, $N^{GBM+MSC}$ = 7, $N^{GBM+MSCMel}$ = 6). One-way ANOVA. (**L**) CD45 and Iba1 immunofluorescence staining of orthotopic tumor sections. The human mitochondria (hMito) marker was used to identify transplanted cells. Scale bar: 100 µm in multicolor images and 500 µm in one color images. Quantification of CD45+ area within the tumor (N^{GBM} = 5, $N^{GBM+MSC}$ = 5, $N^{GBM+MSCMel}$ = 4). Quantification of Iba1+ area within the tumor (N^{GBM} = 5, $N^{GBM+MSC}$ = 5, $N^{GBM+MSCMel}$ = 4). Visual scoring of invading Iba1+ cells around the tumor (N^{GBM} = 5, $N^{GBM+MSC}$ = 5, $N^{GBM+MSCMel}$ = 4). One-way ANOVA. (**M**) Circular heat map of 110 DEG (p-value <0.05) in the comparison GBM+MSC vs. $GBM+MSC^{Mel}$ showing the hierarchical clustering of mRNA expression (log2 fold-change). (**N**) Patient clusters according to non-supervised K-mean clustering method ($N^{cluster\ 1}$=167, $N^{cluster\ 2}$=371). A, astrocytoma; AA, anaplastic astrocytoma; AO, anaplastic oligodendroglioma; AOA, anaplastic oligoastrocytoma; O, oligodendroglioma; OA, oligoastrocytoma. Data are represented as mean ± SEM. *p < 0.05, **p < 0.01, ***p < 0.001, ****p < 0.0001 compared to control group (GBM), unless otherwise indicated.

Table S1. List of primary antibodies.

Antibody	Manufacturer	Cat number	Dilution	Specificity
**BAD**	Cell Signaling (Beverly, MA, USA)	9239S	1:1000	Apoptosis
**Bcl-xL**	Cell Signaling (Beverly, MA, USA)	2764S	1:1000	Apoptosis
**CD105**	BD Biosciences (San Jose, CA, USA)	560839	1:20	MSC characterization
**CD13**	BD Biosciences (San Jose, CA, USA)	347406	1:20	MSC characterization
**CD29**	BD Biosciences (San Jose, CA, USA)	555443	1:20	MSC characterization
**CD31**	BD Biosciences (San Jose, CA, USA)	555445	1:20	MSC characterization
**CD34**	BD Biosciences (San Jose, CA, USA)	555822	1:20	MSC characterization
**CD34**	Abcam (Cambridge, United Kingdom)	ab81289	1:200	Blood vessel cells
**CD45**	BD Biosciences (San Jose, CA, USA)	345808	1:20	MSC characterization
**CD45**	Abcam (Cambridge, United Kingdom)	ab10558	1:200	Immune cells
**CD73**	BD Biosciences (San Jose, CA, USA)	550257	1:20	MSC characterization
**CD90**	BD Biosciences (San Jose, CA, USA)	555595	1:20	MSC characterization
**COL1**	Abcam (Cambridge, United Kingdom)	AB88147	1:200	Extracellular matrix
**COL3**	Abcam (Cambridge, United Kingdom)	AB7778	1:150	Extracellular matrix
**COL4**	Abcam (Cambridge, United Kingdom)	AB6586	1:150	Extracellular matrix
**Cytochrome C**	Cell Signaling (Beverly, MA, USA)	11940S	1:300-1000	Apoptosis
**E-Cadherin**	Cell Signaling (Beverly, MA, USA)	3195S	1:300-1000	EMT Transition
**GAPDH**	Cell Signaling (Beverly, MA, USA)	CST2118	1:30000	House keeping
**GFP**	Thermo (Waltham, MA, USA)	A-11122	1:500	Cell marker
**GM-CSF**	ProteinTech (Rosemont, IL, USA)	177762-1-AP	1:200	Inflammation
**HLA-II**	BD Biosciences (San Jose, CA, USA)	555558	1:20	MSC characterization
**hMito**	Merck Milipore (Molsheim, Francia)	MAB1273	1:100	Human cells
**Iba1**	Wako (Neuss, Germany)	019-19741	1:500	Microglia
**Ki-67**	BD Biosciences (San Jose, CA, USA)	556027	1:20	Proliferation
**Ki-67**	Thermo (Madrid, Spain)	9106-S1	1:200	Proliferation
**m-Cherry**	Thermo (Waltham, MA, USA)	M11217	1:500	Cell marker
**MTNR1A**	Bioss Antibodies (Woburn, MA, USA)	Bs-0027R	1:1000	Mel receptor
**MTNR1B**	Abcam (Cambridge, United Kingdom)	ab203346	1:1000	Mel receptor
**Myosin**	Sigma (St. Louis, MO, USA)	M7648-2ML	1:200	Cytoskeleton
**N-cadherin**	Sigma (St. Louis, MO, USA)	C3865	1:300-1000	Adherent junctions
**Nestin**	Abcam (Cambridge, United Kingdom)	ab6320	1:200	Stemness
**NF-κβ**	Abcam (Cambridge, United Kingdom)	ab32536	1:200	Inflammation
**OCT3/4**	Sigma (St. Louis, MO, USA)	MAB1759	1:500	Stemness
**p16**	Santa Cruz (Heidelberg, Germany)	sc-468	1:1000	Senescence
**p21**	Santa Cruz (Heidelberg, Germany)	sc-6246	1:1000	Senescence
**Phalloidin**	Thermo (Waltham, MA, USA)	A22287	1:200	Cytoskeleton
**pHH3**	Cell Signaling (Beverly, MA, USA)	9701	1:100	Proliferation
**Sox2**	Thermo (Waltham, MA, USA)	14-9811-82	1:150	Stemness
**Vimentin**	Abcam (Cambridge, United Kingdom)	ab1620	1:200-1000	Cytoskeleton
**α-tubulin**	Sigma (St. Louis, MO, USA)	T6199	1:200	Cytoskeleton
**β-actin**	Sigma (St. Louis, MO, USA)	A5441	1:200-1:5000	Cytoskeleton
**β-catenin**	Cell signaling (Beverly, MA, USA)	9561S	1:200	Adherent junctions
**γ-tubulin**	Sigma (St. Louis, MO, USA)	T3320	1:200	Cytoskeleton
